# Updated Swiss Growth References 2025: No Height Differences, but BMI Variations Associated with Migration

**DOI:** 10.3390/jcm14165912

**Published:** 2025-08-21

**Authors:** Urs Eiholzer, Anika Stephan, Ilja Dubinski, Christiane Fritz, Cees Noordam

**Affiliations:** Center for Paediatric Endocrinology Zurich (PEZZ), Moehrlistrasse 69, 8006 Zurich, Switzerland; anika.stephan@pezz.ch (A.S.); ilja.dubinski@pezz.ch (I.D.); chris.fritz@pezz.ch (C.F.); kees.noordam@pezz.ch (C.N.)

**Keywords:** growth references, growth curves, body mass index, percentiles, childhood obesity, public health

## Abstract

**Background/Objectives:** The 2019 Swiss growth references for height, weight, and BMI were based on a large dataset from the German-speaking part of Switzerland (Cohort 2019). The current study aimed to ensure national representativeness by proportionate amounts of additional data from the French-speaking (Suisse Romande) and Italian-speaking (Ticino) regions to validate the 2019 growth curves and to update the national growth references. It also investigated the influence of parental migration background on child growth. **Methods:** Anthropometric data from 43,290 children and adolescents—including 11,816 new cases—were analyzed (Cohort 2019 + 2025). Percentile curves were modeled using the Generalized Additive Models for Location, Scale, and Shape (GAMLSS) framework. **Results:** The extended dataset largely confirms the 2019 growth references. Variations in height percentiles were small and clinically negligible. Clinically relevant differences in BMI percentiles were observed in girls, with the most pronounced deviations—up to 0.8 kg/m^2^—at the 97th percentile. Analyses by parental migration background revealed relevant differences in BMI. **Conclusions:** The extended Swiss Growth References (Cohort 2019 + 2025) are robust and provide valid reference data for all Swiss children and adolescents, offering contemporary tools for decision-making in clinical practice. To maintain their validity over time, targeted updates are required, with special attention to demographic changes resulting from migration.

## 1. Introduction

Growth curves are a fundamental tool for pediatricians, as they provide critical insights into a child’s health and allow for early detection of conditions such as short stature or obesity. The accuracy of these assessments depends on the growth reference used—typically national or regional references, since definitions of abnormal growth should be based on population-specific percentiles [1].

Two major factors necessitate the regular updating of growth references: the secular trend—long-term shifts in growth patterns—and migration, which introduces new genetic influences into the population [2]. Growth references from WHO, CDC, and various European countries provide a global framework for child growth monitoring, yet substantial inter-country differences—driven by genetics, socioeconomic status, and migration—persist. Evidence from Asia, Africa, and North and South America underscores the need for local references to avoid misclassification [1,3,4,5,6,7,8,9,10]. Large-scale European data show that WHO charts often detect fewer children with growth disorders than recent national references, especially in countries with marked secular height gains, whereas older national charts may overestimate short stature. Similar discrepancies have been reported worldwide, with European children often appearing taller and populations from parts of Asia, the Middle East, and Latin America shorter relative to WHO standards, challenging the assumption of a single universal growth curve [1,8]. As a result, numerous countries have developed their own national reference curves [3,4,5,6,7,8,9,10]. Some countries even have two region-specific reference curves to account for internal diversity (e.g., Italy and Turkey) [11,12]. Thus, the ‘one size fits all’ approach of the WHO has been largely abandoned.

Switzerland, with a total area of 41,285 km^2^—70% of which is uninhabited—is a small country both geographically and demographically. It comprises four linguistic regions, three of which border countries with the same national language: the German-speaking north (~5.5 million people), the French-speaking west (~2 million), and the Italian-speaking south (~350,000). The fourth, Romansh-speaking region (~40,000) is limited to parts of eastern Graubünden [13]. In 2019, we published new reference curves for Switzerland (Cohort 2019) based on data collected mainly in the German-speaking part of Switzerland. Our analysis demonstrated that these national reference curves more accurately reflect the growth patterns of Swiss children than the WHO curves, which have been in use from 2011 to the present [14,15,16]. Prior to the WHO standards, the reference curves of the ‘First Zurich Longitudinal Study’, also known as the ‘Prader curves’, were used in Switzerland [17]. Notably, both the WHO and Prader curves are based on cohorts born in the 1950s, but, unsurprisingly, the ‘Prader curves’ already better reflected the reality in Switzerland than the WHO curves [14]. The closest alignment of our 2019 curves was with those of our neighboring countries Germany and Austria. In addition, we examined weight and BMI depending on migration status and found that immigration from Southern Europe accounts for the main part of the increase in BMI and the prevalence of obesity in Switzerland [18].

Following discussions within the Swiss Pediatric Society (‘pädiatrie schweiz’), the inclusion of all linguistic regions of Switzerland in a national growth reference was requested—primarily to reflect regional representativeness. This position was taken despite the fact that data from Swiss military recruits indicate an interregional difference in final height of only 0.6 cm [19]. While a north–south gradient in height and BMI is well established across Europe, with taller and leaner children in the north and shorter, heavier children in the south [4,20,21,22,23,24]—such patterns are less likely to be observed in a geographically compact and socioeconomically homogeneous country like Switzerland [14,20,21,22,23,24].

**Figure 1:**
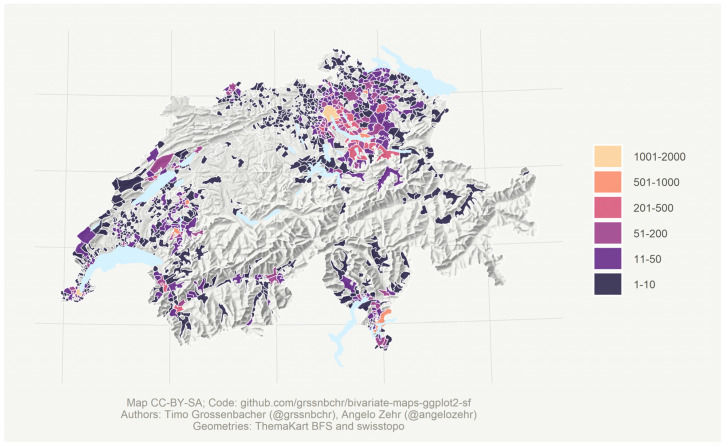
Map of Switzerland showing the geographical distribution and number of children and adolescents measured in the current study (Cohort 2019 + 2025) by municipality of residence. The map illustrates the spatial coverage of the cohort (residential information was only available for measurements from pediatricians’ practices, schools, and vocational schools; for newborns and recruits, information was only available at the cantonal level and is not displayed here).

Nevertheless, the Swiss Pediatric Society insisted on additional data from other regions of Switzerland before the data from the Cohort 2019 could be considered as the new “Swiss reference”. To support broad acceptance, we expanded our Cohort 2019 study by collecting new data in the French-speaking western region (Romandie) and the Italian-speaking southern region (Ticino) following the same protocol as in 2019, creating the Cohort 2019 + 2025. The number of records collected per municipality is illustrated in Figure 1. As migration flows vary in different linguistic regions, this update additionally could provide a better understanding of how migration affects height and weight. Our former study [18] has demonstrated that the increase in child obesity in Switzerland is primarily attributable to children with parents originating from Southern Europe. Additionally, other studies have highlighted that pubertal timing, including the onset and progression of the growth spurt, can vary by ethnicity [25,26,27].

The combined dataset (Cohort 2019 + 2025) allows for a more comprehensive analysis of growth patterns in Switzerland and the influence of migration. Our hypothesis was that height would remain comparable across regions without differences compared to the original study and that there could be differences in overweight prevalences based upon local differences in composition of the population regarding ethnicity due to migration.

## 2. Materials and Methods

### 2.1. Study Design and Sample

The prospectively collected cross-sectional sample of the 2019 study and the data of the Zurich school medical service were re-used. Our study used anonymously collected health-related data, which does not fall under the Swiss Human Research Act. The local ethics committee confirmed it was outside their jurisdiction and ethically harmless, involving no risk or more than minimal burden (Cantonal Ethics Committee Zurich/“Kantonale Ethikkommission Zürich”, declaration of non-objection BASEC-Nr. Req-2017-00440). In pediatric practices, parents received information leaflets and provided verbal consent. School measurements were conducted with approval from the school and education department; parents were informed via leaflets, and project staff presented oral information to each class, explaining the right to dissent. Data privacy was ensured by conducting measurements individually and apart from classmates. For details of the anthropometric measurements, the ethics committee waiver, and consent procedures, see also Eiholzer et al. 2019 [14].

In 2023, additional cross-sectional data were prospectively collected with the same protocol from 10 pediatric practices located in the Italian-speaking part of Switzerland (Ticino), and in 2024/2025 in 34 pediatric practices and two secondary schools located in the French-speaking part of Switzerland (Suisse Romande). For a visual illustration of the different data origins by municipality, please see Figure 1. Children who had undergone pediatric consultation for growth problems or had a disease affecting growth (e.g., celiac disease, renal insufficiency, or hormonal disorders) were excluded.

Retrospective data of the school medical services (collection years 2020 to 2024) were provided from three communities of Suisse Romande (Bulle, Biel, and Geneve). Exclusion criteria in this data subset could not be ascertained due to the lack of available medical information.

Data of all births registered in Switzerland from 2017 to 2021 were provided by the Federal Office for Population Statistics (BEVNAT). We excluded births of pre- and late-term children (gestational age < 39 weeks or >41 weeks) and used stratified random drawing (strata: year, sex, and canton) to obtain 3000 births, representative of births in Switzerland.

Data of recruits from the Swiss military medical service were provided for the years 2018 to 2022. We filtered for age < 21.5 in males and age < 19.5 in females. We used stratified random drawing to obtain data of a total of 100 females (strata: year and canton) and 3495 males (strata: age, year, and canton).

After data cleaning procedures, our sample included a total of 23,768 boys and 19,522 girls (Table 1). There were 24,893 records re-used from the 2019 collection in German-speaking Switzerland, 2337 were collected in Ticino, 9479 were collected in Suisse Romande, and 6581 records were extracted from national data sets (BEVNAT, Swiss military medical service).

The categorization of migration groups was applied as described before [14]. Table 2 shows the distributions of parental origin for the Cohort 2019 + 2025 data set. Of note, parental origin is not part of the retrospective data (newborns, military recruits, and school medical service).

### 2.2. Data Handling and Statistical Analysis

Data cleaning included checking for duplicates, obvious input errors, and typical input errors such as confusing weight with height or errors in the decimal place. Data were checked for outliers by applying different outlier definitions from sex- and age-specific percentiles. Height values lying outside the interval [P25 − 4.5(P50 − P25); P75 + 4.5(P75 − P50)] and weight values lying outside the interval [P25 − 5(P50 − P25)^0.75^; P75 + 9(P75 − P50)] were defined as outliers, and the respective cases were excluded (*n* = 132).

Growth curve modelling was performed using the Generalized Additive Models for Location, Scale, and Shape (GAMLSS) framework with the Box–Cox Cole and Green original (BCCGo) distribution. GAMLSS encompasses several distributions for growth data, including the classic Lambda–Mu–Sigma (LMS) method described by Cole and Green [28]. Within the GAMLSS framework, the LMS method corresponds to the BCCGo distribution, which models three age-dependent parameters: μ (median), σ (coefficient of variation), and ν (Box–Cox power, representing skewness) and typically provides an adequate fit for human growth data [29].

As a preparatory step, we fitted a base model using the lms() function with age transformation and cubic penalized splines (with smoothing parameter k = 3 or k = log(*n*)) to determine an appropriate level of smoothness and inform spline complexity for the final models. This step also ensured monotonicity and a biologically plausible shape of the median curve.

We then developed two height models per sex: one for early infancy (m1, age < 2 years) and one for later childhood (m2, age > 0.25 years), each with tailored smoothing for the location and scale parameters. These models were combined at 1.2 years. Monotonic behavior in the 3rd, 50th, and 97th percentiles was enforced by applying pbm() smoothing to the location term and adjusting scale smoothing accordingly. Overfitting was minimized by inspecting velocity curves to avoid unrealistic fluctuations. Model diagnostics were evaluated using worm plots, excluding the newborn period due to the disproportionately high case numbers and potential measurement error.

Weight was modelled following a similar approach. Model m1 was fitted for ages below 5 years, and model m2 for ages above 3 years; the two models were joined at 4.25 years. Age transformation was applied only in model m1. For BMI, a single model was used for ages above 1.5 years. Non-monotonic behavior was allowed in the BMI models; age was transformed for boys but left untransformed for girls.

We graphically compared the newly estimated percentiles (3rd, 50th, and 97th) with their corresponding values from the Cohort 2019 and calculated absolute differences. Discrepancies were considered noticeable if differences exceeded 0.5 cm for height or 0.3 kg/m^2^ for BMI. In such cases, we examined potential regional influences by analyzing the distribution of data points from the three regional collections (German-speaking Switzerland, Suisse Romande, and Ticino) within the percentile and age range of interest using the Chi-square test and evaluating the test’s standardized residuals.

Prevalence of overweight, including obesity, and of obesity alone, were calculated using the International Obesity Task Force (IOTF) cut-offs. These cutoffs are defined by the percentiles at BMI values of 25 kg/m^2^ and 30 kg/m^2^ at the age of 18 years throughout childhood and adolescence and were derived from six large data sets from Brazil, Great Britain, Hong Kong, the Netherlands, Singapore, and the United States [30]. We also applied the IOTF method on our data to obtain our own empirically determined cut-off percentiles.

Additionally, we calculated the distributions of origin within the total sample and within individuals categorized as overweight (including obese) and obese based on the IOTF classification. We examined whether height-for-age or BMI-for-age percentiles are influenced by the high proportion of foreigners and second-generation immigrants in Switzerland. Therefore, we defined subgroups containing children with parents (1) both from Switzerland, (2) both from Northern/Central Europe, (3) both from Italy, Spain, or Portugal, (4) both from the Balkan region, (5) both from Turkey, or (6) both from other regions or (7) both from different regions. Additionally, we evaluated a group with (8) only one parent with Swiss origin.

All statistical analyses were conducted in R (version 4.4.1). We used the following R packages: tidyverse (v2.0.0; [31]), gamlss (v5.4–22; [32]), sitar (v1.4.0; [33]), and qqplotr (v0.0.6).

## 3. Results

### 3.1. Height

**Figure 2:**
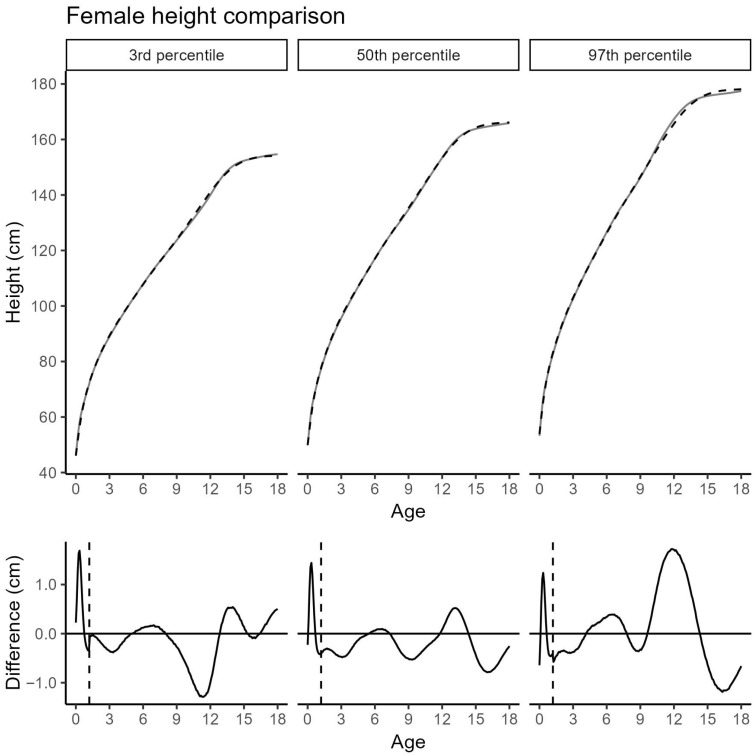
Comparison of the 3rd, 50th, and 97th female height percentiles between the Cohort 2019 (black, dashed) and the extended Cohort 2019 + 2025 (grey, solid). (**Top**): absolute height; (**bottom**): difference (Cohort 2019 + 2025 minus Cohort 2019); vertical dashed line: age transition between the models; black solid line: difference in cm between the two cohorts.

The updated growth curves differ only slightly from the previous Cohort 2019 across most percentiles and age groups [14]. In girls, deviations in the 3rd and 50th percentiles, as well as in the 97th percentile outside of puberty remain within ±0.5 cm. The most notable deviation occurs in girls around the pubertal age of 12 years, where the 97th percentile is elevated by 1.7 cm (Figure 2). Additionally, records from Ticino were overrepresented at or above the 97th percentile for girls between 9 and 13 years of age (Chi-square statistic = 6.0914; *p* = 0.048).

**Figure 3:**
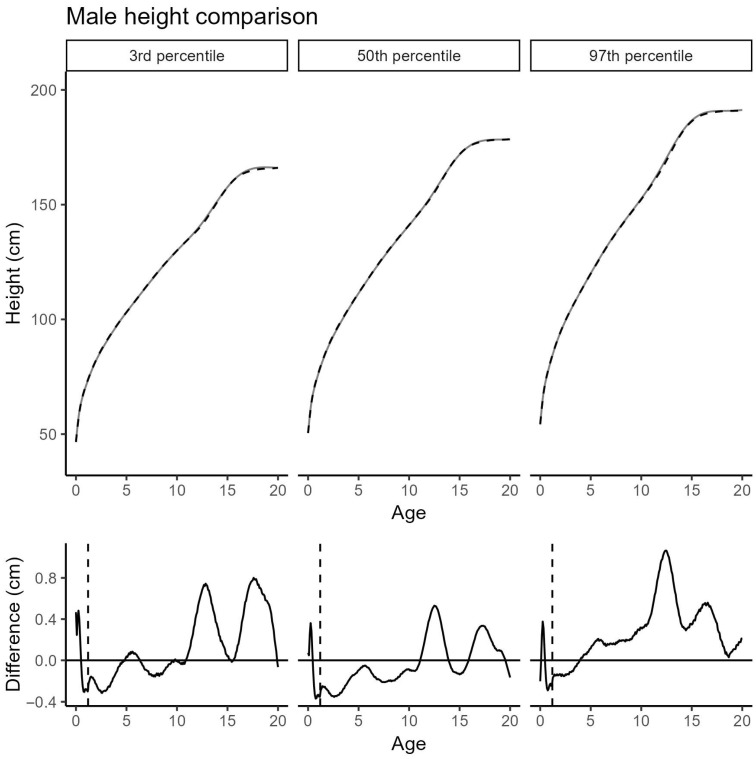
Comparison of the 3rd, 50th, and 97th male height percentiles between the Cohort 2019 (black, dashed) and the extended Cohort 2019 + 2025 (grey, solid). (**Top**): absolute height; (**bottom**): difference (Cohort 2019 + 2025 minus Cohort 2019); vertical dashed line: age transition between the models; black solid line: difference in cm between the two cohorts.

In boys, the updated curves show minimal deviations: at the 3rd and 50th percentiles as well as across most ages within the 97th percentile, differences remain within ±0.8 cm. A subtle spike-like deviation at around 3 months—similar to that observed in girls—is attributed to over-smoothing in the 2019 model and has now been corrected. The largest deviation appears at the 97th percentile around age 12, where height is increased by 1.0 cm compared to the previous curves (Figure 3).

Overall, the revised curves exhibit strong agreement with the previous model across the full age range in both sexes. Corresponding height values for key percentiles are provided in Table 3, with further details, including model parameters, available in Supplementary Tables S1 and S2.

### 3.2. BMI

**Figure 4:**
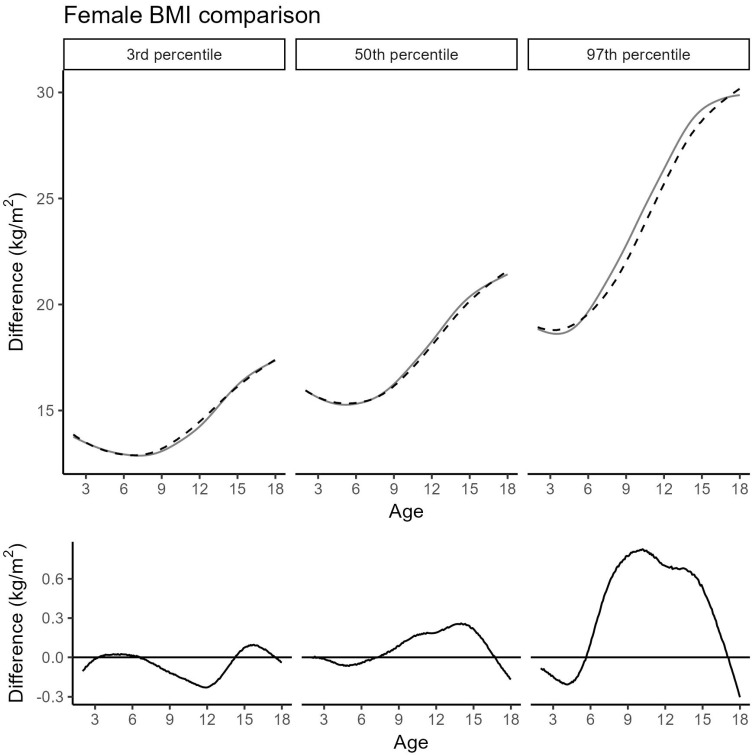
Comparison of the 3rd, 50th, and 97th female BMI percentiles between the Cohort 2019 (black, dashed) and the extended Cohort 2019 + 2025 (grey, solid). (**Top**): absolute BMI; (**bottom**): difference (Cohort 2019 + 2025 minus Cohort 2019).

**Figure 5:**
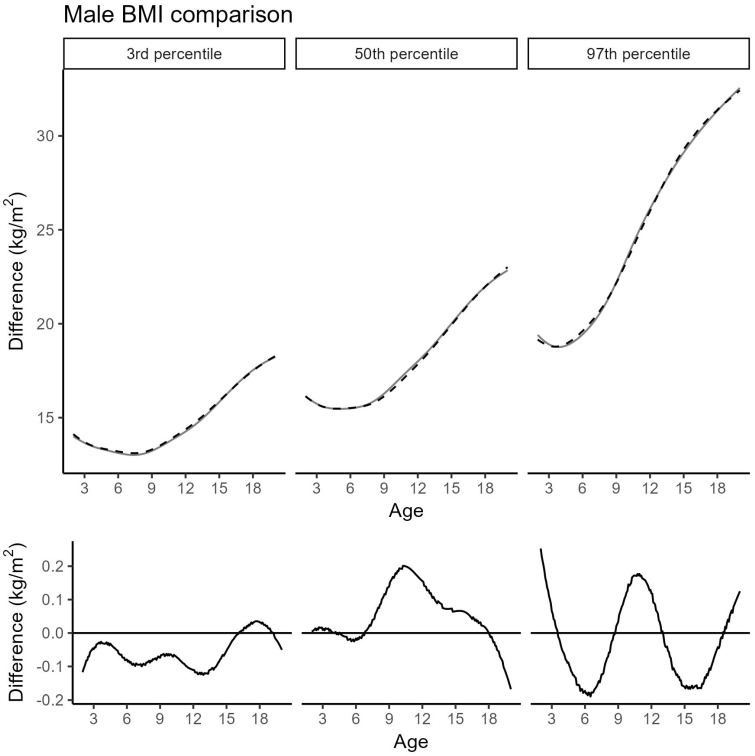
Comparison of the 3rd, 50th, and 97th male BMI percentiles between the Cohort 2019 (black, dashed) and the extended Cohort 2019 + 2025 (grey, solid). (**Top**): absolute BMI; (**bottom**): difference (Cohort 2019 + 2025 minus Cohort 2019).

Figure 4 and Figure 5 show the updated BMI percentiles for girls and boys, respectively. Across all percentiles for boys and in the 3rd and 50th percentiles for girls, deviations from the 2019 cohort remained minimal and did not exceed 0.3 kg/m^2^. More pronounced differences were found in the 97th percentile for girls between 8 and 15 years of age, with deviations reaching up to 0.8 kg/m^2^. Within this subgroup, data from French-speaking Switzerland were significantly overrepresented, while data from German-speaking Switzerland were significantly underrepresented (*p* = 0.011; Table 4). Corresponding BMI values for selected percentiles are listed in Table 5. Further model details are provided in Supplementary Tables S3 and S4; percentiles for weight and weight-for-height appear in Supplementary Tables S5–S8.

### 3.3. Cut-Off Values for Overweight and Obesity

For girls, the BMI percentiles corresponding to overweight and obesity cut-offs (defined as BMI values of 25 and 30 kg/m^2^ at age 18 years) calculated from the Cohort 2019 + 2025 are the 84.3rd and 97.1st percentiles, respectively. For boys, the corresponding percentiles are 79.1 and 95.6. These percentiles are included in the Supplementary Tables S3 and S4.

### 3.4. Parental Origin Across Swiss Regions

The distribution of parental origin varied across the three collection regions (Table 2). Suisse Romande and Ticino had fewer children with both parents from Switzerland and more with both parents from Italy/Spain/Portugal. German-speaking Switzerland had more children with both parents from the Balkan region and Turkey, and Ticino showed fewer children with both parents from Northern/Central Europe.

### 3.5. Overweight and Obesity by Parental Origin

Obesity, as defined by IOTF [30], showed marked variation by parental origin (Table 6). Prevalence was below 2% among children with both parents from Switzerland or Northern/Central Europe but reached approximately 6% in girls with parental origin in the Balkan region or Italy/Spain/Portugal and up to 8% in boys from the Balkan region. In the total sample, obesity was observed in 2.8% of girls and 3% of boys.

A similar but less pronounced pattern emerged for overweight: overall, 13.1% of girls and 14% of boys were affected. Among children with both parents from Switzerland or Northern/Central Europe, prevalence was lower—around 10% and 7%, respectively. In contrast, overweight affected about 20% of children whose parents came from Italy, Spain, or Portugal. Girls with parental origin in the Balkan region or Turkey also showed rates around 20%, with even higher rates among boys from these backgrounds (approximately 26%).

**Figure 6:**
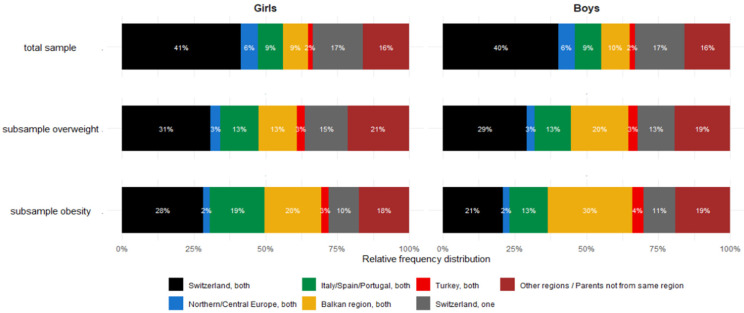
Distribution of parental origin among the total Cohort 2019 + 2025 and among children classified as overweight (including obesity) and obese according to International Obesity Task Force (IOTF) cut-off criteria [30]. Analysis restricted to age ≥2 years.

Thus, certain migration groups are disproportionately represented in the overweight and obese categories relative to their share in the total cohort (Figure 6). Although girls from the ‘Italy/Spain/Portugal’ and ‘Balkan’ groups make up together 18% of the total Cohort 2019 + 2025, they account for 26% of all overweight (including obesity) and 39% of all obesity cases. Among boys, these two groups represent 19% of the total cohort together but 33% of all overweight and 43% of all obesity cases. The inverse is true for girls with both parents of Swiss origin: while their share in all girls is 41%, they constitute only 31% of the overweight and 28% of the obesity group. For boys with Swiss parents, the pattern is similar.

## 4. Discussion

In this study, we present new reference values for height, weight, weight for height, and BMI in children currently living in Switzerland, based on data collected across the three major linguistic regions. These new percentiles are compared with recently established reference data from the largest region, the German-speaking part of Switzerland [14].

The extended dataset (Cohort 2019 + 2025) confirms the robustness of the 2019 Swiss growth references for height, weight, and BMI [14]. This is not unexpected, given that (1) Switzerland is a relatively small country with a population of approximately 9 million [13]; (2) the similarity of the growth curves to those of the neighboring countries such as Germany and Austria has been previously demonstrated [14]; and (3) data from Swiss recruits indicate that regional height differences across linguistic areas are well under 1.0 cm [19].

Switzerland has the second-highest proportion of immigrants in Europe (27%), most of whom come from other European countries. These populations are unevenly distributed across the country’s three main linguistic regions. The extension of the dataset to include all three regions was intended to improve representativeness and account for potential demographic differences. While regional differences in growth patterns were overall minimal, immigration emerged as the most influential factor.

Our most important finding was a clinically relevant shift in the upper female BMI percentiles, primarily driven by data from Suisse Romande. This pattern is likely linked to regional variation in the composition and concentration of immigrant groups. In contrast, height percentiles and male BMI percentiles showed only minor deviations.

### 4.1. Height

For clinical diagnostic purposes—particularly in prepubertal age groups (3–9 years for girls and 3–11 years for boys)—the 3rd percentile is the most relevant, as it is primarily within this age range that the differential diagnosis of short stature is most frequently considered. The differences here are less than ±0.4 cm. Around the age of pubertal development, the differences become more pronounced—reaching up to 1.3 cm in girls and 0.8 cm in boys.

Compared to the 2019 cohort [14], we observe a larger deviation in the 3rd and in the 97th percentiles for girls in the pubertal age range. This likely reflects a greater proportion of shorter girls (e.g., with delayed growth) and taller girls (e.g., with early maturation) in the expanded dataset. The most pronounced deviations occurred around the onset of puberty—a known period of high variability influenced by ethnicity [25,26]. This interpretation is supported by the overrepresentation of girls from Ticino in the ≥97th percentiles between ages 9 and 13. Compared to other regions, Ticino included fewer children with Swiss or Northern/Central European parental origin and more with parents from Italy, Spain, or Portugal. These differences in ethnic composition likely contributed to regional variation in pubertal timing and thus to the observed percentile shifts—an expected pattern in cross-sectional data [34].

Switching from a one-part to a two-part age model altered percentile shapes. The current model showed spike-like artifacts at the age of 3 months in both sexes—likely due to excessive smoothing during rapid postnatal growth. The current two-part model allows more flexibility and offers a more accurate reflection of true growth patterns, without indicating real differences between the cohorts at that age.

To what extent must growth curves be precise to effectively support clinical decision-making? The central issue here is clinical relevance. Christesen et al. [1] showed that WHO reference thresholds may delay the diagnosis of growth disorders in European children. For example, a deviation of up to 4 cm at the 3rd percentile in prepubertal children, when compared to WHO standards, can correspond to a delay in height development of approximately one year [14]. In our own 2019 study on Swiss children, we found a comparable discrepancy: the 3rd percentile of the WHO reference curve lay up to 4 cm below our study, particularly between the ages of 7 and 16 years [14].

### 4.2. BMI

At the age of 10–12 years, a BMI difference of 1 kg/m^2^ at the 50th percentile corresponds to a weight difference of approximately 2 kg. Accordingly, a deviation of 0.3 kg/m^2^ represents a weight difference of roughly 670 g, which falls within the range of normal circadian weight fluctuations and is therefore not clinically relevant [35]. This applies to both boys and girls at the 3rd and 50th percentiles, where the observed deviations remain minor. Clinically relevant differences begin at ≥0.5 kg/m^2^—a threshold clearly exceeded by many children with a migration background from Southern and Southeastern Europe in Switzerland. The work of Cole et al. [30] revealed that national BMI curves vary by ±1.0 kg/m^2^ for genetically distant populations (e.g., Netherlands vs. Brazil). Accordingly, a difference below 0.3 kg/m^2^ for all boys’ percentiles and for the lower and middle girls’ percentiles between the Cohort 2019 and the Cohort 2019 + 2025 is small.

However, the 97th percentile for girls within the age range of 8 to 15 years was significantly elevated in the Cohort 2019 + 2025. The maximum difference of 0.8 kg/m^2^ translates to a weight difference of about 1.6 kg for a 12-year-old girl of median height. This exceeds normal daily variation and may be clinically relevant. For boys, however, the corresponding deviation at the 97th percentile is smaller and remains within the range of physiological weight variation.

The 90th and 97th percentiles do not constitute an appropriate definition of overweight and obesity, with observed deviations ranging from +1.0 to +2.0 kg/m^2^. Overweight, as defined by IOTF [30], was observed in 13.1% of the girls of the extended Cohort 2019 + 2025 and obesity in 2.8%. These values were 14% and 3% for the boys, respectively (Table 6). These findings of proportions of overweight and obesity of Cohort 2019 + 2025 were slightly smaller than reported from Cohort 2019 alone [14].

### 4.3. Disproportionate Burden of Overweight and Obesity by Country of Origin

The BMI data from the extended Cohort 2019 + 2025 confirm the findings reported in 2019 and 2021 [14,18]: overweight and obesity in Switzerland is not proportionally distributed across the evaluated groups of parental origin but is concentrated in specific subgroups (Figure 6), particularly among children with both parents from Southern Europe and the Balkan region [18,36].

Among children with obesity, the overrepresentation of these two origin groups is striking—they account for 29% of obese girls and 43% of obese boys, despite constituting less than 20% of the overall sample. Similarly, they represent up to 26% of overweight girls and 33% of overweight boys (overweight including obesity). Therefore, understanding the underlying causes of this disproportionate prevalence of unhealthy weight in these subpopulations is essential for addressing and reducing health inequalities within the Swiss pediatric population.

### 4.4. Factors Influencing Growth Curve Updates and Limitations

Several additional considerations, including both contextual factors and methodological limitations, are relevant when interpreting these findings.

Although socioeconomic status was not directly assessed, previous analyses of the Cohort 2019 dataset have shown that ethnic origin has a stronger influence on overweight and obesity risk than socioeconomic factors, suggesting a predominant role of genetic and cultural components in these disparities [18].

The most significant difference—and likely the strongest determinant of BMI and potentially the timing of pubertal development and growth spurts—is the differing composition of groups by country of origin. Immigration patterns shaped by linguistic ties to neighboring countries result in contrasting effects: children of Southern European origin tend toward smaller stature and higher BMI, while those of Northern European origin tend toward taller height and lower BMI.

To maintain the clinical validity of growth references in a country with high migration and population turnover such as Switzerland, targeted and timely updates would be helpful. In this context, migration patterns—characterized by regional clustering and rapid change—are more relevant than long-term secular trends and should be a central focus. Instead of relying on longitudinal data collection, we advocate a pragmatic approach: regularly reassessing specific segments of the growth curves with stratification by migration background. This targeted monitoring allows for more responsive adjustments to changing population dynamics. In the future, advanced modeling techniques—such as synthetic growth curves or big-data-driven individualization—may help to generate personalized growth references that integrate both genetic and demographic factors [37,38].

This study is based on cross-sectional data, which does not allow causal conclusions for specific growth phases such as the pubertal spurt. While the sample is large and includes children and adolescents in proportional numbers from all three major language regions of Switzerland, some selection bias may remain, particularly for migrant subgroups that are underrepresented. Migration background was grouped into simplified categories that may not fully capture cultural or socioeconomic diversity; for example, in French-speaking Switzerland, the category “Italy, Spain, Portugal” may contain a higher proportion of Portuguese participants than the corresponding group in Ticino. Anthropometric measurements were taken using standardized protocols, but some inter-observer variation cannot be excluded. The analysis was limited to height, weight, and BMI; additional anthropometric measures such as waist circumference or body composition were not available, which could have provided further insight into observed BMI differences. Additionally, the military datasets used in this study have inherent limitations, as height was routinely rounded to the nearest centimeter and weight often to the nearest kilogram; while such measurement imprecision may not fully meet the standards of scientific anthropometric assessment, the large sample sizes are likely to have minimized the impact of random measurement error.

## 5. Conclusions

The findings of this study demonstrate that the 2019 Swiss growth curves [14] remain robust and valid across all major linguistic regions of Switzerland. The extended dataset (Cohort 2019 + 2025) confirms the stability of the height references and reinforces their clinical applicability. Observed differences in BMI percentiles are mainly driven by shifts in migrant population composition rather than intrinsic biological variations within the native Swiss population, highlighting the impact of demographic changes on growth. The disproportionate prevalence of overweight and obesity among children from Southern and Southeastern Europe underscores significant health disparities. This also emphasizes the importance of considering migration background and ethnicity in growth assessments. Practically, the Cohort 2019 + 2025 growth curves provide an updated reference for clinical and public health use throughout Switzerland.

Ongoing surveillance and updates of growth references remain necessary due to demographic changes. Future research should explore advanced modelling techniques, including synthetic growth curves and big-data approaches, to enable personalized growth assessments integrating genetic, environmental, and sociodemographic factors. Switzerland combines a very small and well-defined territory with complete migration statistics and clearly traceable migration flows. This combination allows analyses that are difficult to achieve in larger nations and makes Switzerland a possible model for studying migration-related growth patterns.

## Figures and Tables

**Table 1:** Number of cases for girls and boys per data source by 2-year increment age groups.

	Girls				Boys			
	Prospective				Prospective			
Age Group (Years)	Pediatric Practice	Schools	Retrospective	Total	Pediatric Practice	Schools	Retrospective	Total
<2.00	3953	0	1475 ^a^	5428	3920	0	1511 ^a^	5431
2–3.99	1393	4	0	1397	1457	4	2 ^b^	1463
4–5.99	1274	2	1768 ^b^	3044	1475	3	1927 ^b^	3405
6–7.99	1046	198	405 ^b^	1649	1082	207	487 ^b^	1776
8–9.99	704	302	78 ^b^	1084	660	321	84 ^b^	1065
10–11.99	1096	93	1254 ^b^	2443	1126	106	1267 ^b^	2499
12–13.99	693	261	411 ^b^	1365	716	210	387 ^b^	1313
14–15.99	605	447	391 ^b^	1443	647	442	430 ^b^	1519
16–17.99	213	840	24 ^b^	1077	219	838	16 ^b^	1073
18–19.99	25	466	100 ^c^	591	32	627	2495 ^c^	3154
20–21.99	1	0	0	1	4	66	1000 ^c^	1070
Total	11,003	2613	5906	19,522	11,338	2824	9606	23,768

^a^ Data source: births. ^b^ Data source: school medical service. ^c^ Data source: military recruits.

**Table 2:** Parental origin, overall and by region of data collection.

Characteristic (For Data from Pediatric Practices and Schools)	Overall (Cohort 2019 + 2025) *n* = 43,290 ^1^	German-Speaking Switzerland *n* = 29,827 ^1^	Suisse Romande *n* = 10,889 ^1^	Ticino *n* = 2574 ^1^
Switzerland, both	12,791 (42%)	9305 (46%)	2763 (34%)	723 (30%)
Northern/Central Europe, both	1698 (5.5%)	1154 (5.7%)	532 (6.5%)	12 (0.5%)
Italy/Spain/Portugal, both	2665 (8.7%)	1147 (5.7%)	968 (12%)	550 (23%)
Balkan region, both	2915 (9.5%)	2048 (10%)	645 (7.9%)	222 (9.1%)
Turkey, both	499 (1.6%)	422 (2.1%)	55 (0.7%)	22 (0.9%)
other regions, both	3095 (10%)	1855 (9.2%)	1055 (13%)	185 (7.6%)
non-Swiss regions, mixed	1726 (5.6%)	848 (4.2%)	666 (8.2%)	212 (8.7%)
Switzerland, one	5375 (17%)	3389 (17%)	1483 (18%)	503 (21%)
Unknown (for retrospective data: newborns, military recruits, and school medical service)	12,526	9659	2722	145

^1^ *n* (%), percentages are based on the part of the sample where parental origin is known (pediatric practices and schools).

**Table 3:** Smoothed height percentiles (in cm) for girls and boys from the Cohort 2019 + 2025.

	Girls							Boys						
Age (Years)	3rd	10th	25th	50th	75th	90th	97th	3rd	10th	25th	50th	75th	90th	97th
0	46.3	47.4	48.5	49.7	51.0	52.1	53.2	47.0	48.1	49.3	50.5	51.8	53.0	54.2
0.25	55.6	57.0	58.3	59.8	61.3	62.6	64.0	57.1	58.4	59.8	61.2	62.7	64.1	65.5
0.5	61.6	63.1	64.6	66.2	67.9	69.4	70.9	63.6	65.0	66.3	67.9	69.5	70.9	72.3
0.75	65.7	67.2	68.8	70.5	72.3	73.9	75.5	67.7	69.1	70.6	72.3	74.0	75.5	77.0
1	69.3	71.0	72.6	74.5	76.3	78.0	79.7	71.1	72.7	74.3	76.0	77.8	79.5	81.1
1.25	72.9	74.5	76.2	78.0	79.9	81.6	83.3	74.3	75.9	77.6	79.5	81.4	83.1	84.9
1.5	75.9	77.6	79.3	81.2	83.2	85.0	86.7	77.2	78.9	80.6	82.6	84.7	86.5	88.3
1.75	78.6	80.3	82.1	84.1	86.2	88.0	89.9	79.8	81.6	83.4	85.5	87.6	89.5	91.5
2	81.0	82.8	84.7	86.8	88.9	90.8	92.8	82.2	84.0	86.0	88.1	90.3	92.3	94.3
3	89.0	91.1	93.2	95.7	98.1	100.4	102.6	90.1	92.3	94.4	96.9	99.4	101.7	103.9
4	95.7	98.1	100.6	103.3	106.1	108.6	111.2	97.0	99.3	101.7	104.4	107.2	109.7	112.2
5	102.0	104.6	107.3	110.3	113.4	116.2	119.0	103.1	105.7	108.4	111.4	114.5	117.2	120.0
6	107.9	110.8	113.7	117.1	120.5	123.5	126.6	108.8	111.7	114.7	118.0	121.4	124.5	127.5
7	113.5	116.6	119.8	123.4	127.1	130.4	133.7	114.5	117.5	120.7	124.3	127.9	131.2	134.5
8	118.6	121.9	125.3	129.1	132.9	136.4	139.9	120.0	123.2	126.5	130.2	133.9	137.4	140.8
9	123.7	127.2	130.7	134.8	138.8	142.6	146.3	125.2	128.5	131.9	135.7	139.6	143.2	146.7
10	128.8	132.6	136.6	141.0	145.5	149.5	153.6	130.0	133.4	137.0	141.0	145.1	148.9	152.6
11	134.1	138.3	142.5	147.3	152.2	156.7	161.1	134.5	138.2	142.1	146.5	150.9	154.9	159.0
12	140.0	144.2	148.5	153.4	158.3	162.8	167.3	139.3	143.4	147.7	152.5	157.4	161.9	166.3
13	146.3	150.2	154.3	158.9	163.5	167.7	171.9	145.1	149.6	154.2	159.4	164.6	169.5	174.3
14	150.4	154.1	157.9	162.2	166.6	170.5	174.5	151.6	156.2	160.9	166.2	171.6	176.5	181.5
15	152.4	156.0	159.7	163.8	168.0	171.9	175.7	157.6	162.1	166.7	171.8	177.1	181.9	186.7
16	153.3	156.9	160.5	164.6	168.8	172.5	176.3	162.2	166.4	170.8	175.6	180.6	185.1	189.6
17	154.1	157.7	161.3	165.3	169.4	173.1	176.8	165.0	168.9	173.0	177.6	182.2	186.4	190.7
18	154.7	158.2	161.8	165.9	170.0	173.7	177.4	166.1	169.9	173.8	178.3	182.7	186.8	190.9
19								166.3	170.1	174.0	178.4	182.8	186.9	190.9
20								166.0	169.9	173.9	178.4	182.9	187.1	191.3

**Table 4:** Proportion of data sets of girls per collection region for the age range 8 to 15 years for all BMI percentiles versus within the 97th to 100th percentile.

	Girls Aged 8 to 15 Years		
Region	All Percentiles	97th–100th	Residual in 97th–100th
German-speaking Switzerland	2346 (64%)	50 (49%)	−3.0 *
Suisse Romande	971 (26%)	38 (38%)	2.5 *
Ticino	373 (10%)	14 (14%)	1.2
Chi-square statistic	9.0664; *p* = 0.011		

* A residual above +2 or below −2 typically indicates a statistically significant overrepresentation or underrepresentation, respectively.

**Table 5:** Smoothed BMI percentiles (in kg/m^2^) for girls and boys from the Cohort 2019 + 2025.

	Girls							Boys						
Age (Years)	3rd	10th	25th	50th	75th	90th	97th	3rd	10th	25th	50th	75th	90th	97th
2	13.8	14.4	15.1	16.0	16.9	17.8	18.9	14.0	14.6	15.3	16.1	17.1	18.2	19.4
3	13.5	14.1	14.8	15.6	16.6	17.6	18.7	13.7	14.2	14.9	15.7	16.7	17.7	18.9
4	13.2	13.8	14.5	15.4	16.4	17.4	18.7	13.4	14.0	14.7	15.5	16.5	17.5	18.8
5	13.1	13.7	14.4	15.3	16.4	17.5	19.0	13.3	13.9	14.6	15.5	16.5	17.6	19.0
6	12.9	13.6	14.3	15.3	16.5	17.9	19.7	13.1	13.8	14.5	15.5	16.7	17.9	19.4
7	12.9	13.6	14.4	15.5	16.9	18.5	20.6	13.0	13.7	14.5	15.6	16.9	18.3	20.1
8	12.9	13.7	14.6	15.8	17.3	19.2	21.7	13.0	13.8	14.7	15.8	17.3	18.9	21.0
9	13.1	13.9	14.9	16.2	18.0	20.0	22.8	13.2	14.0	15.0	16.3	17.9	19.8	22.2
10	13.4	14.3	15.4	16.9	18.8	21.0	24.0	13.5	14.4	15.4	16.8	18.6	20.7	23.6
11	13.8	14.8	15.9	17.5	19.6	22.0	25.2	13.9	14.8	15.9	17.4	19.4	21.7	24.9
12	14.3	15.3	16.6	18.3	20.4	23.0	26.4	14.3	15.2	16.4	18.0	20.1	22.6	26.1
13	14.9	16.0	17.3	19.0	21.3	24.0	27.5	14.7	15.7	16.9	18.6	20.8	23.5	27.2
14	15.6	16.7	18.0	19.8	22.1	24.8	28.5	15.2	16.3	17.5	19.3	21.6	24.3	28.2
15	16.2	17.3	18.6	20.4	22.7	25.4	29.2	15.8	16.9	18.2	20.0	22.3	25.2	29.1
16	16.7	17.8	19.0	20.8	23.1	25.8	29.6	16.5	17.6	18.9	20.7	23.1	26.0	29.9
17	17.1	18.1	19.4	21.1	23.4	26.1	29.8	17.0	18.2	19.5	21.4	23.8	26.7	30.7
18	17.4	18.4	19.7	21.4	23.7	26.3	29.9	17.5	18.7	20.1	22.0	24.4	27.3	31.3
19								17.9	19.1	20.5	22.4	24.9	27.9	32.0
20								18.2	19.4	20.9	22.8	25.4	28.4	32.6

**Table 6:** IOTF-based rates of overweight and obesity (in percent) according to parent origin (analysis restricted to age ≥ 2 years).

	Cohort 2019 + 2025					
	Girls			Boys		
	*n*	Overweight	Obese	*n*	Overweight	Obese
Switzerland, both	4009	9.8	1.9	4108	10.2	1.6
Northern/Central Europe, both	576	7.5	1.2	567	7.2	1.2
Italy/Spain/Portugal, both	835	20.1	6.1	933	19.1	4.4
Balkan region, both	826	20.2	6.4	1055	27.2	8.5
Turkey, both	165	21.8	4.2	176	25.6	6.2
Switzerland, one	1696	11.1	1.7	1770	10.6	2.1
Other regions/Parents not from the same region	1555	17.4	3.0	1629	16.9	3.7
Total	9662	13.1	2.8	10,238	14.0	3.0

## Data Availability

Data supporting the findings of this study are available from the corresponding author upon request.

## Supplementary Materials

The following supporting information can be downloaded at https://www.mdpi.com/article/10.3390/jcm14165912/s1, Table S1: Smoothed height percentiles (in cm) and model parameters for girls aged 0 to 18 years; Table S2: Smoothed height percentiles (in cm) and model parameters for boys aged 0 to 20 years; Table S3: Smoothed BMI percentiles (in kg/m^2^) and model parameters for girls aged 2 to 18 years; Table S4: Smoothed BMI percentiles (in kg/m^2^), cut-off values, and model parameters for boys aged 2 to 20 years; Table S5: Smoothed weight percentiles (in kg) and model parameters for girls aged 0 to 18 years; Table S6: Smoothed weight percentiles (in kg) and model parameters for boys aged 0 to 20 years; Table S7: Smoothed weight (kg) for height (cm) percentiles and model parameters for girls; Table S8: Smoothed weight (kg) for height (cm) percentiles and model parameters for boys. Figure S1: Density histograms of standardized height data. Figure S2: Density histograms of standardized BMI data. Dashed line: calculated cut-off for overweight, dot-dashed line: calculated cut-off for obesity (see Section 3.3). Figure S3: Density histograms of standardized weight data. Text S1–S4: Participating pediatricians and schools.

## Abbreviations

The following abbreviations are used in this manuscript:

BEVNAT	German: Statistik der natürlichen Bevölkerungsbewegung
BMI	Body Mass Index
BCCGo	Box–Cox Cole and Green original distribution
CDC	Centers for Disease Control and Prevention
Cohort 2019	Cohort studied in 2019 and published here: [14]
Cohort 2019 + 2025	Extended current dataset, which includes additional proportional samples from the French- and Italian-speaking parts of Switzerland
GAMLSS	Generalized Additive Models for Location, Scale, and Shape
IOTF	International Obesity Task Force
LMS	Lambda–Mu–Sigma method for growth curve modelling
WHO	World Health Organization
