# Supplementary material for: Updated Swiss Growth References 2025: No Height Differences, but BMI Variations Associated with Migration
Source: J Clin Med. 2025 Aug 21;14(16):5912. doi: 10.3390/jcm14165912 (PMC12387566; doi:10.3390/jcm14165912)
Supplement: Supplementary file 1 [file jcm-14-05912-s001.zip › jcm-3796415 Supplemental material to Manuscript Cohort 2019+2025 Supp. Table S1-S8 Figure S1-S3. (Edited).pdf]

Supplemental Material, Table S1–S8 and Figure S1–S3

Table S1: Smoothed **height** percentiles (in cm) and model parameters for **girls** aged 0 to 18 years.

	Percentiles							Model parameters	
years	3	10	25	50*	75	90	97	L	S
0	46.284	47.379	48.491	49.732	50.977	52.102	53.215	0.8513	0.0371
0.083	49.458	50.627	51.816	53.142	54.472	55.674	56.864	0.8513	0.0371
0.167	52.711	53.957	55.224	56.637	58.055	59.336	60.604	0.8513	0.0371
0.25	55.638	56.954	58.291	59.782	61.279	62.631	63.970	0.8513	0.0371
0.333	58.085	59.459	60.855	62.412	63.974	65.386	66.783	0.8513	0.0371
0.417	60.036	61.456	62.899	64.508	66.123	67.582	69.026	0.8513	0.0371
0.5	61.635	63.092	64.574	66.226	67.884	69.382	70.865	0.8513	0.0371
0.583	63.045	64.536	66.051	67.741	69.437	70.969	72.486	0.8513	0.0371
0.667	64.366	65.888	67.436	69.161	70.892	72.456	74.005	0.8513	0.0371
0.75	65.654	67.206	68.784	70.544	72.310	73.906	75.485	0.8513	0.0371
0.833	66.911	68.494	70.102	71.895	73.695	75.321	76.931	0.8513	0.0371
0.917	68.134	69.745	71.383	73.209	75.042	76.698	78.337	0.8513	0.0371
1	69.317	70.956	72.623	74.480	76.345	78.030	79.697	0.8513	0.0371
1.083	70.457	72.123	73.817	75.705	77.601	79.313	81.008	0.8513	0.0371
1.167	71.552	73.243	74.963	76.881	78.806	80.545	82.266	0.8513	0.0371
1.25	72.925	74.534	76.181	78.029	79.897	81.595	83.287	0.5515	0.0353
1.333	73.950	75.583	77.254	79.130	81.026	82.750	84.468	0.5515	0.0353
1.417	74.936	76.594	78.291	80.195	82.120	83.869	85.613	0.5515	0.0354
1.5	75.887	77.570	79.292	81.225	83.178	84.955	86.724	0.5515	0.0355
1.583	76.805	78.513	80.260	82.222	84.205	86.008	87.804	0.5515	0.0356
1.667	77.692	79.425	81.198	83.189	85.201	87.030	88.853	0.5515	0.0357
1.75	78.550	80.308	82.107	84.126	86.167	88.023	89.873	0.5515	0.0358
1.833	79.381	81.164	82.988	85.036	87.106	88.989	90.865	0.5515	0.0359
1.917	80.186	81.993	83.843	85.919	88.019	89.928	91.830	0.5515	0.0360
2	80.967	82.799	84.674	86.778	88.906	90.841	92.770	0.5515	0.0362
2.5	85.231	87.207	89.229	91.499	93.795	95.884	97.966	0.5515	0.0370
3	88.962	91.078	93.245	95.678	98.140	100.380	102.613	0.5515	0.0379
3.5	92.404	94.658	96.967	99.562	102.188	104.577	106.959	0.5515	0.0389
4	95.727	98.115	100.562	103.313	106.097	108.630	111.158	0.5515	0.0397
4.5	98.915	101.431	104.008	106.906	109.839	112.510	115.174	0.5515	0.0404
5	101.965	104.602	107.304	110.342	113.418	116.219	119.014	0.5515	0.0411
5.5	104.950	107.712	110.542	113.725	116.949	119.885	122.815	0.5515	0.0418
6	107.894	110.785	113.749	117.082	120.459	123.535	126.605	0.5515	0.0425
6.5	110.762	113.775	116.865	120.341	123.863	127.072	130.275	0.5515	0.0431
7	113.519	116.640	119.840	123.441	127.090	130.415	133.734	0.5515	0.0435
7.5	116.137	119.346	122.638	126.343	130.097	133.517	136.933	0.5515	0.0438
8	118.642	121.929	125.301	129.095	132.940	136.443	139.942	0.5515	0.0439
8.5	121.133	124.507	127.967	131.861	135.807	139.404	142.995	0.5515	0.0441
9	123.674	127.159	130.735	134.759	138.839	142.557	146.270	0.5515	0.0446
9.5	126.244	129.878	133.606	137.803	142.059	145.939	149.814	0.5515	0.0455
10	128.821	132.640	136.560	140.975	145.453	149.537	153.618	0.5515	0.0468

years	Percentiles 3	10	25	50*	75	90	97	Model parameters L	S
10.5	131.423	135.431	139.547	144.184	148.890	153.182	157.474	0.5515	0.0480
11	134.094	138.250	142.518	147.328	152.210	156.664	161.118	0.5515	0.0488
11.5	136.923	141.152	145.495	150.389	155.356	159.888	164.419	0.5515	0.0486
12	140.010	144.218	148.537	153.404	158.340	162.843	167.344	0.5515	0.0474
12.5	143.257	147.359	151.568	156.306	161.110	165.489	169.863	0.5515	0.0453
13	146.258	150.221	154.284	158.855	163.486	167.704	171.916	0.5515	0.0429
13.5	148.649	152.483	156.411	160.828	165.300	169.372	173.435	0.5515	0.0410
14	150.393	154.123	157.944	162.239	166.585	170.540	174.486	0.5515	0.0395
14.5	151.583	155.242	158.988	163.198	167.457	171.332	175.196	0.5515	0.0385
15	152.351	155.967	159.668	163.827	168.033	171.860	175.675	0.5515	0.0379
15.5	152.880	156.466	160.137	164.261	168.432	172.226	176.008	0.5515	0.0374
16	153.336	156.894	160.535	164.625	168.761	172.523	176.272	0.5515	0.0370
16.5	153.765	157.296	160.909	164.967	169.071	172.802	176.522	0.5515	0.0367
17	154.149	157.662	161.258	165.296	169.379	173.092	176.792	0.5515	0.0364
17.5	154.462	157.976	161.572	165.610	169.692	173.404	177.104	0.5515	0.0363
18	154.666	158.200	161.816	165.876	169.982	173.716	177.437	0.5515	0.0365

*50th percentile = model parameter M

Table S2: Smoothed **height** percentiles (in cm) and model parameters for **boys** aged 0 to 20 years.

years	Percentiles 3	10	25	50*	75	90	97	Model parameters L	S
0	47.017	48.123	49.257	50.531	51.821	52.996	54.168	0.5192	0.0376
0.083	50.404	51.613	52.851	54.243	55.653	56.937	58.218	0.5192	0.0383
0.167	53.951	55.207	56.494	57.940	59.404	60.737	62.067	0.5192	0.0372
0.25	57.148	58.438	59.758	61.241	62.742	64.108	65.471	0.5192	0.0361
0.333	59.785	61.097	62.440	63.948	65.473	66.861	68.245	0.5192	0.0352
0.417	61.865	63.196	64.559	66.089	67.637	69.044	70.447	0.5192	0.0345
0.5	63.599	64.953	66.340	67.896	69.470	70.901	72.328	0.5192	0.0342
0.583	65.099	66.480	67.894	69.481	71.086	72.546	74.000	0.5192	0.0341
0.667	66.444	67.856	69.300	70.922	72.562	74.053	75.539	0.5192	0.0341
0.75	67.701	69.144	70.622	72.281	73.958	75.483	77.004	0.5192	0.0342
0.833	68.905	70.381	71.893	73.590	75.307	76.868	78.424	0.5192	0.0344
0.917	70.054	71.565	73.111	74.848	76.604	78.202	79.795	0.5192	0.0346
1	71.144	72.689	74.272	76.048	77.846	79.480	81.110	0.5192	0.0348
1.083	72.179	73.759	75.377	77.195	79.033	80.706	82.373	0.5192	0.0351
1.167	73.174	74.791	76.445	78.304	80.184	81.894	83.600	0.5192	0.0354
1.25	74.255	75.895	77.577	79.469	81.385	83.131	84.874	0.4620	0.0355
1.333	75.254	76.923	78.635	80.560	82.511	84.288	86.062	0.4620	0.0357
1.417	76.218	77.916	79.657	81.615	83.599	85.407	87.211	0.4620	0.0358
1.5	77.150	78.876	80.645	82.635	84.652	86.489	88.324	0.4620	0.0359
1.583	78.052	79.804	81.601	83.623	85.671	87.538	89.401	0.4620	0.0361

years	Percentiles							Model parameters	
	3	10	25	50*	75	90	97	L	S
1.667	78.924	80.703	82.527	84.579	86.659	88.554	90.446	0.4620	0.0362
1.75	79.769	81.574	83.424	85.506	87.616	89.539	91.458	0.4620	0.0363
1.833	80.588	82.418	84.294	86.405	88.544	90.494	92.441	0.4620	0.0365
1.917	81.382	83.236	85.137	87.277	89.445	91.421	93.395	0.4620	0.0366
2	82.152	84.030	85.956	88.123	90.320	92.322	94.321	0.4620	0.0367
2.5	86.372	88.382	90.443	92.764	95.116	97.260	99.402	0.4620	0.0373
3	90.136	92.261	94.441	96.895	99.384	101.652	103.918	0.4620	0.0378
3.5	93.643	95.875	98.166	100.745	103.359	105.744	108.126	0.4620	0.0382
4	96.987	99.329	101.733	104.441	107.186	109.690	112.192	0.4620	0.0387
4.5	100.153	102.617	105.146	107.995	110.885	113.522	116.157	0.4620	0.0394
5	103.128	105.727	108.397	111.404	114.456	117.241	120.025	0.4620	0.0403
5.5	105.996	108.736	111.550	114.722	117.942	120.882	123.821	0.4620	0.0413
6	108.832	111.706	114.658	117.987	121.368	124.454	127.542	0.4620	0.0422
6.5	111.650	114.639	117.711	121.176	124.695	127.908	131.123	0.4620	0.0427
7	114.451	117.531	120.696	124.265	127.891	131.202	134.515	0.4620	0.0429
7.5	117.226	120.375	123.610	127.259	130.966	134.350	137.737	0.4620	0.0428
8	119.960	123.163	126.454	130.165	133.935	137.377	140.821	0.4620	0.0426
8.5	122.621	125.874	129.216	132.984	136.811	140.306	143.802	0.4620	0.0423
9	125.180	128.488	131.886	135.718	139.609	143.162	146.716	0.4620	0.0422
9.5	127.628	131.006	134.476	138.390	142.363	145.991	149.621	0.4620	0.0422
10	129.966	133.439	137.008	141.032	145.119	148.852	152.586	0.4620	0.0426
10.5	132.226	135.825	139.524	143.697	147.936	151.808	155.683	0.4620	0.0434
11	134.476	138.233	142.096	146.454	150.884	154.932	158.984	0.4620	0.0445
11.5	136.793	140.736	144.791	149.369	154.025	158.280	162.542	0.4620	0.0458
12	139.284	143.421	147.678	152.487	157.379	161.853	166.335	0.4620	0.0472
12.5	142.060	146.376	150.820	155.840	160.950	165.624	170.309	0.4620	0.0482
13	145.113	149.572	154.163	159.352	164.633	169.466	174.310	0.4620	0.0487
13.5	148.332	152.879	157.562	162.853	168.239	173.167	178.107	0.4620	0.0486
14	151.593	156.163	160.868	166.184	171.592	176.540	181.498	0.4620	0.0478
14.5	154.749	159.283	163.948	169.216	174.574	179.473	184.381	0.4620	0.0466
15	157.646	162.095	166.669	171.833	177.081	181.876	186.678	0.4620	0.0449
15.5	160.169	164.499	168.949	173.969	179.067	183.724	188.384	0.4620	0.0431
16	162.248	166.447	170.759	175.620	180.554	185.058	189.563	0.4620	0.0413
16.5	163.839	167.909	172.088	176.795	181.571	185.928	190.284	0.4620	0.0398
17	164.968	168.927	172.989	177.563	182.202	186.432	190.658	0.4620	0.0385
17.5	165.700	169.573	173.546	178.019	182.553	186.686	190.815	0.4620	0.0375
18	166.104	169.925	173.843	178.254	182.723	186.797	190.866	0.4620	0.0369
18.5	166.264	170.064	173.961	178.346	182.790	186.841	190.886	0.4620	0.0367
19	166.262	170.070	173.976	178.371	182.826	186.886	190.940	0.4620	0.0368
19.5	166.160	170.003	173.945	178.381	182.877	186.975	191.069	0.4620	0.0371
20	165.990	169.887	173.884	178.383	182.945	187.104	191.258	0.4620	0.0377

*50th percentile = model parameter M

Table S3: Smoothed **BMI** percentiles (in kg/m²) and model parameters for **girls** aged 2 to 18 years.

	**Percentiles**									**Model parameters**	
**years**	**3**	**10**	**25**	**50***	**75**	**90**	**97**	***OW***	***OB***	**L**	**S**
2	13.763	14.398	15.097	15.949	16.890	17.827	18.847	*17.4*	*18.9*	-0.7942	0.0831
2.5	13.613	14.233	14.921	15.768	16.716	17.671	18.728	*17.2*	*18.8*	-0.9929	0.0841
3	13.475	14.083	14.763	15.608	16.566	17.546	18.646	*17.1*	*18.7*	-1.1792	0.0853
3.5	13.348	13.948	14.625	15.474	16.449	17.460	18.614	*17.0*	*18.6*	-1.3464	0.0870
4	13.233	13.831	14.510	15.371	16.372	17.424	18.647	*16.9*	*18.7*	-1.4922	0.0892
4.5	13.133	13.734	14.422	15.303	16.341	17.449	18.761	*16.9*	*18.8*	-1.6190	0.0923
5	13.050	13.660	14.363	15.273	16.360	17.541	18.968	*17.0*	*19.0*	-1.7287	0.0961
5.5	12.984	13.606	14.330	15.278	16.427	17.699	19.273	*17.1*	*19.3*	-1.8220	0.1007
6	12.932	13.572	14.322	15.315	16.537	17.917	19.667	*17.3*	*19.7*	-1.8929	0.1059
6.5	12.894	13.554	14.335	15.378	16.681	18.178	20.124	*17.5*	*20.2*	-1.9298	0.1116
7	12.872	13.557	14.372	15.470	16.856	18.474	20.617	*17.7*	*20.7*	-1.9274	0.1173
7.5	12.875	13.589	14.442	15.598	17.069	18.803	21.131	*18.0*	*21.2*	-1.8875	0.1229
8	12.909	13.656	14.551	15.768	17.324	19.168	21.660	*18.3*	*21.7*	-1.8176	0.1283
8.5	12.977	13.761	14.702	15.984	17.626	19.575	22.212	*18.6*	*22.3*	-1.7304	0.1334
9	13.081	13.904	14.894	16.244	17.973	20.024	22.793	*19.0*	*22.9*	-1.6381	0.1382
9.5	13.220	14.084	15.124	16.543	18.359	20.510	23.404	*19.5*	*23.5*	-1.5525	0.1426
10	13.387	14.293	15.382	16.868	18.770	21.017	24.029	*19.9*	*24.1*	-1.4776	0.1465
10.5	13.571	14.516	15.653	17.204	19.186	21.522	24.640	*20.4*	*24.7*	-1.4120	0.1498
11	13.770	14.752	15.934	17.544	19.600	22.017	25.230	*20.8*	*25.3*	-1.3574	0.1525
11.5	13.992	15.008	16.230	17.894	20.017	22.508	25.810	*21.3*	*25.9*	-1.3175	0.1545
12	14.245	15.290	16.547	18.259	20.441	23.001	26.388	*21.8*	*26.5*	-1.2962	0.1557
12.5	14.539	15.606	16.892	18.643	20.876	23.497	26.969	*22.2*	*27.1*	-1.2968	0.1560
13	14.868	15.952	17.257	19.036	21.310	23.984	27.538	*22.7*	*27.6*	-1.3201	0.1554
13.5	15.217	16.309	17.624	19.421	21.722	24.438	28.068	*23.1*	*28.2*	-1.3645	0.1539
14	15.567	16.660	17.977	19.780	22.094	24.839	28.533	*23.5*	*28.6*	-1.4252	0.1518
14.5	15.899	16.986	18.298	20.096	22.410	25.168	28.907	*23.8*	*29.0*	-1.4947	0.1491
15	16.199	17.277	18.580	20.366	22.672	25.429	29.192	*24.1*	*29.3*	-1.5641	0.1464
15.5	16.460	17.530	18.822	20.595	22.887	25.635	29.401	*24.3*	*29.5*	-1.6239	0.1437
16	16.684	17.747	19.030	20.792	23.068	25.801	29.555	*24.5*	*29.7*	-1.6707	0.1414
16.5	16.879	17.937	19.214	20.965	23.228	25.944	29.676	*24.6*	*29.8*	-1.7037	0.1394
17	17.051	18.107	19.380	21.124	23.375	26.071	29.769	*24.8*	*29.9*	-1.7224	0.1377
17.5	17.209	18.264	19.536	21.274	23.512	26.185	29.832	*24.9*	*29.9*	-1.7267	0.1362
18	17.360	18.417	19.688	21.422	23.646	26.289	29.875	*25.0*	*30.0*	-1.7200	0.1347

*50th percentile = model parameter M, OW: cut-off for overweight, OB: cut-off for obesity

Table S4: Smoothed **BMI** percentiles (in kg/m[2]), cut-off values, and model parameters for **boys** aged 2 to 20 years.

	Percentiles									Model parameters	
years	3	10	25	50*	75	90	97	*OW*	*OB*	L	S
2	14.004	14.604	15.284	16.145	17.143	18.192	19.407	*17.4*	*19.0*	-1.5861	0.0849
2.5	13.822	14.411	15.079	15.923	16.902	17.929	19.117	*17.1*	*18.7*	-1.5861	0.0844
3	13.658	14.242	14.903	15.739	16.708	17.725	18.903	*16.9*	*18.5*	-1.5861	0.0845
3.5	13.523	14.105	14.767	15.604	16.575	17.597	18.783	*16.8*	*18.4*	-1.5861	0.0854
4	13.418	14.005	14.673	15.520	16.507	17.549	18.761	*16.7*	*18.4*	-1.5861	0.0871
4.5	13.335	13.933	14.614	15.481	16.496	17.571	18.831	*16.7*	*18.4*	-1.5861	0.0895
5	13.261	13.873	14.574	15.469	16.523	17.647	18.974	*16.8*	*18.6*	-1.5861	0.0928
5.5	13.187	13.817	14.542	15.473	16.576	17.763	19.177	*16.8*	*18.7*	-1.5861	0.0967
6	13.119	13.769	14.521	15.494	16.655	17.916	19.436	*16.9*	*19.0*	-1.5861	0.1012
6.5	13.061	13.735	14.516	15.534	16.761	18.106	19.748	*17.0*	*19.2*	-1.5861	0.1061
7	13.023	13.720	14.533	15.600	16.896	18.333	20.109	*17.2*	*19.5*	-1.5861	0.1111
7.5	13.014	13.735	14.582	15.699	17.069	18.605	20.529	*17.4*	*19.9*	-1.5861	0.1162
8	13.046	13.793	14.674	15.846	17.294	18.935	21.020	*17.6*	*20.4*	-1.5861	0.1211
8.5	13.120	13.895	14.812	16.040	17.571	19.324	21.580	*17.9*	*20.9*	-1.5861	0.1258
9	13.234	14.037	14.993	16.279	17.896	19.766	22.206	*18.3*	*21.4*	-1.5861	0.1304
9.5	13.378	14.211	15.206	16.552	18.256	20.247	22.878	*18.7*	*22.0*	-1.5861	0.1346
10	13.543	14.404	15.438	16.843	18.635	20.747	23.570	*19.1*	*22.6*	-1.5861	0.1385
10.5	13.718	14.607	15.677	17.139	19.016	21.245	24.258	*19.5*	*23.3*	-1.5861	0.1420
11	13.894	14.809	15.914	17.429	19.384	21.723	24.916	*19.8*	*23.9*	-1.5861	0.1450
11.5	14.073	15.012	16.149	17.712	19.739	22.178	25.535	*20.2*	*24.4*	-1.5861	0.1476
12	14.261	15.222	16.388	17.995	20.086	22.615	26.118	*20.6*	*25.0*	-1.5861	0.1496
12.5	14.466	15.448	16.640	18.288	20.436	23.042	26.669	*20.9*	*25.5*	-1.5861	0.1510
13	14.696	15.698	16.915	18.598	20.797	23.469	27.199	*21.3*	*26.0*	-1.5861	0.1518
13.5	14.955	15.975	17.216	18.932	21.175	23.903	27.714	*21.7*	*26.4*	-1.5861	0.1521
14	15.236	16.275	17.537	19.283	21.564	24.337	28.208	*22.1*	*26.9*	-1.5861	0.1519
14.5	15.532	16.588	17.870	19.643	21.955	24.763	28.675	*22.5*	*27.4*	-1.5861	0.1513
15	15.837	16.909	18.209	20.005	22.344	25.178	29.115	*22.9*	*27.8*	-1.5861	0.1504
15.5	16.145	17.232	18.550	20.366	22.728	25.581	29.532	*23.3*	*28.2*	-1.5861	0.1493
16	16.451	17.552	18.885	20.720	23.101	25.970	29.927	*23.7*	*28.6*	-1.5861	0.1481
16.5	16.748	17.862	19.209	21.062	23.461	26.343	30.303	*24.0*	*29.0*	-1.5861	0.1470
17	17.030	18.157	19.518	21.387	23.802	26.698	30.662	*24.4*	*29.4*	-1.5861	0.1459
17.5	17.292	18.431	19.806	21.691	24.124	27.034	31.005	*24.7*	*29.7*	-1.5861	0.1450
18	17.530	18.680	20.068	21.970	24.421	27.348	31.336	*25.0*	*30.0*	-1.5861	0.1444
18.5	17.741	18.902	20.304	22.223	24.694	27.642	31.654	*25.0*	*30.0*	-1.5861	0.1439
19	17.926	19.099	20.513	22.450	24.943	27.917	31.961	*25.0*	*30.0*	-1.5861	0.1438
19.5	18.088	19.272	20.700	22.655	25.172	28.175	32.259	*25.0*	*30.0*	-1.5861	0.1439
20	18.232	19.426	20.868	22.842	25.385	28.420	32.551	*25.0*	*30.0*	-1.5861	0.1441

*50th percentile = model parameter M, OW: cut-off for overweight, OB: cut-off for obesity

Table S5: Smoothed **weight** percentiles (in kg) and model parameters for **girls** aged 0 to 18 years.

	Percentiles							Model parameters	
**years**	**3**	**10**	**25**	**50***	**75**	**90**	**97**	**L**	**S**
0	2.658	2.870	3.094	3.357	3.633	3.893	4.161	0.3668	0.1189
0.083	3.302	3.560	3.835	4.157	4.497	4.818	5.149	0.3342	0.1179
0.167	3.945	4.249	4.574	4.954	5.356	5.737	6.131	0.3042	0.1170
0.25	4.554	4.900	5.270	5.705	6.164	6.601	7.053	0.2753	0.1161
0.333	5.095	5.477	5.886	6.367	6.876	7.361	7.864	0.2471	0.1153
0.417	5.543	5.953	6.392	6.910	7.459	7.982	8.526	0.2192	0.1144
0.5	5.895	6.326	6.787	7.331	7.910	8.462	9.037	0.1917	0.1135
0.583	6.186	6.632	7.110	7.675	8.277	8.852	9.452	0.1645	0.1127
0.667	6.462	6.921	7.415	8.000	8.623	9.220	9.843	0.1375	0.1119
0.75	6.744	7.218	7.728	8.332	8.978	9.597	10.245	0.1108	0.1111
0.833	7.023	7.511	8.037	8.661	9.329	9.970	10.642	0.0842	0.1105
0.917	7.287	7.788	8.329	8.972	9.661	10.323	11.019	0.0578	0.1099
1	7.528	8.042	8.597	9.256	9.965	10.648	11.366	0.0316	0.1095
1.083	7.748	8.273	8.840	9.516	10.243	10.944	11.684	0.0055	0.1092
1.167	7.947	8.481	9.060	9.751	10.495	11.215	11.975	-0.0205	0.1090
1.25	8.133	8.677	9.267	9.972	10.734	11.471	12.251	-0.0464	0.1089
1.333	8.320	8.874	9.476	10.197	10.976	11.733	12.535	-0.0721	0.1090
1.417	8.522	9.088	9.703	10.441	11.242	12.020	12.846	-0.0978	0.1091
1.5	8.736	9.315	9.946	10.703	11.526	12.328	13.182	-0.1234	0.1093
1.583	8.950	9.542	10.188	10.966	11.812	12.639	13.521	-0.1488	0.1096
1.667	9.148	9.752	10.413	11.210	12.079	12.930	13.840	-0.1742	0.1100
1.75	9.326	9.942	10.616	11.432	12.323	13.197	14.134	-0.1995	0.1105
1.833	9.489	10.116	10.803	11.635	12.548	13.445	14.408	-0.2247	0.1110
1.917	9.641	10.278	10.978	11.828	12.760	13.680	14.670	-0.2499	0.1115
2	9.786	10.433	11.145	12.011	12.964	13.905	14.922	-0.2750	0.1120
2.5	10.646	11.353	12.139	13.107	14.188	15.274	16.465	-0.4242	0.1156
3	11.614	12.388	13.257	14.342	15.572	16.828	18.231	-0.5716	0.1192
3.5	12.118	12.925	13.843	15.001	16.336	17.723	19.302	-0.7174	0.1226
4	13.131	14.006	15.010	16.294	17.799	19.391	21.245	-0.8619	0.1261
4.5	13.956	14.900	15.993	17.403	19.077	20.872	22.997	-0.9372	0.1304
5	14.755	15.781	16.976	18.535	20.407	22.446	24.900	-0.9910	0.1361
5.5	15.544	16.664	17.979	19.713	21.823	24.160	27.025	-1.0325	0.1432
6	16.318	17.545	18.998	20.934	23.326	26.020	29.393	-1.0599	0.1516
6.5	17.100	18.441	20.043	22.199	24.897	27.983	31.923	-1.0695	0.1601
7	17.918	19.379	21.133	23.511	26.513	29.984	34.473	-1.0581	0.1673
7.5	18.787	20.375	22.287	24.890	28.193	32.033	37.028	-1.0270	0.1734
8	19.716	21.443	23.526	26.365	29.975	34.174	39.641	-0.9797	0.1788
8.5	20.714	22.595	24.868	27.967	31.905	36.480	42.419	-0.9218	0.1839
9	21.787	23.845	26.331	29.721	34.022	39.006	45.444	-0.8583	0.1892
9.5	22.949	25.204	27.928	31.640	36.339	41.763	48.728	-0.7923	0.1944
10	24.199	26.672	29.657	33.716	38.837	44.716	52.204	-0.7234	0.1993
10.5	25.534	28.242	31.504	35.924	41.470	47.786	55.743	-0.6494	0.2032
11	26.958	29.910	33.457	38.242	44.205	50.933	59.309	-0.5785	0.2061

	Percentiles							Model parameters	
**years**	**3**	**10**	**25**	**50***	**75**	**90**	**97**	**L**	**S**
11.5	28.491	31.683	35.507	40.644	47.006	54.128	62.907	-0.5234	0.2076
12	30.184	33.587	37.654	43.102	49.821	57.304	66.471	-0.4970	0.2072
12.5	32.070	35.630	39.881	45.566	52.568	60.357	69.886	-0.5073	0.2044
13	34.093	37.748	42.109	47.943	55.132	63.142	72.969	-0.5506	0.1994
13.5	36.160	39.846	44.243	50.129	57.396	65.521	75.542	-0.6204	0.1925
14	38.160	41.821	46.187	52.033	59.270	67.393	77.476	-0.7092	0.1844
14.5	39.984	43.581	47.868	53.612	60.735	68.762	78.786	-0.8070	0.1759
15	41.544	45.064	49.256	54.874	61.850	69.737	79.638	-0.9022	0.1682
15.5	42.798	46.251	50.362	55.872	62.724	70.493	80.292	-0.9859	0.1621
16	43.751	47.165	51.231	56.686	63.483	71.213	81.011	-1.0512	0.1583
16.5	44.467	47.872	51.930	57.380	64.183	71.942	81.814	-1.0934	0.1563
17	45.025	48.449	52.529	58.009	64.854	72.666	82.620	-1.1087	0.1555
17.5	45.485	48.946	53.068	58.597	65.488	73.334	83.297	-1.0950	0.1552
18	45.885	49.400	53.575	59.158	66.082	73.915	83.780	-1.0536	0.1549

*50th percentile = model parameter M

Table S6: Smoothed **weight** percentiles (in kg) and model parameters for **boys** aged 0 to 20 years.

	Percentiles							Model parameters	
**years**	**3**	**10**	**25**	**50***	**75**	**90**	**97**	**L**	**S**
0	2.779	2.997	3.227	3.495	3.775	4.039	4.310	0.4026	0.1163
0.083	3.415	3.720	4.039	4.403	4.777	5.123	5.473	0.6612	0.1244
0.167	4.233	4.597	4.976	5.411	5.860	6.274	6.694	0.6304	0.1210
0.25	4.916	5.318	5.740	6.225	6.727	7.194	7.669	0.5535	0.1176
0.333	5.476	5.904	6.354	6.876	7.420	7.929	8.450	0.4575	0.1149
0.417	5.932	6.377	6.848	7.397	7.974	8.518	9.077	0.3555	0.1129
0.5	6.320	6.778	7.266	7.839	8.444	9.018	9.613	0.2555	0.1114
0.583	6.663	7.132	7.636	8.229	8.861	9.465	10.093	0.1612	0.1104
0.667	6.969	7.449	7.966	8.580	9.237	9.868	10.530	0.0738	0.1097
0.75	7.247	7.738	8.268	8.901	9.583	10.241	10.935	-0.0060	0.1094
0.833	7.508	8.009	8.554	9.206	9.912	10.597	11.325	-0.0785	0.1092
0.917	7.761	8.274	8.832	9.504	10.235	10.949	11.710	-0.1439	0.1093
1	8.006	8.529	9.103	9.795	10.552	11.294	12.088	-0.2026	0.1095
1.083	8.237	8.773	9.361	10.073	10.855	11.625	12.453	-0.2551	0.1098
1.167	8.453	9.000	9.602	10.334	11.141	11.938	12.799	-0.3019	0.1101
1.25	8.656	9.214	9.830	10.581	11.411	12.235	13.128	-0.3434	0.1105
1.333	8.849	9.418	10.047	10.817	11.671	12.520	13.445	-0.3804	0.1110
1.417	9.036	9.616	10.259	11.048	11.924	12.800	13.756	-0.4134	0.1114
1.5	9.220	9.811	10.468	11.275	12.175	13.077	14.064	-0.4427	0.1119
1.583	9.401	10.004	10.675	11.501	12.424	13.351	14.369	-0.4688	0.1124
1.667	9.580	10.194	10.879	11.724	12.670	13.622	14.671	-0.4922	0.1129
1.75	9.756	10.382	11.081	11.944	12.913	13.891	14.971	-0.5130	0.1134

	Percentiles							Model parameters	
**years**	**3**	**10**	**25**	**50***	**75**	**90**	**97**	**L**	**S**
1.833	9.929	10.567	11.280	12.162	13.154	14.157	15.267	-0.5316	0.1139
1.917	10.099	10.749	11.476	12.376	13.391	14.419	15.559	-0.5484	0.1144
2	10.264	10.925	11.666	12.585	13.622	14.675	15.844	-0.5635	0.1148
2.5	11.121	11.846	12.662	13.681	14.841	16.029	17.362	-0.6297	0.1176
3	11.912	12.697	13.585	14.700	15.978	17.296	18.785	-0.6743	0.1202
3.5	12.721	13.567	14.528	15.739	17.135	18.583	20.232	-0.7139	0.1222
4	13.601	14.509	15.543	16.853	18.369	19.952	21.764	-0.7529	0.1237
4.5	14.421	15.401	16.527	17.972	19.672	21.478	23.590	-0.8631	0.1289
5	15.242	16.295	17.517	19.104	20.999	23.047	25.491	-0.9556	0.1340
5.5	16.070	17.202	18.528	20.269	22.379	24.702	27.534	-1.0372	0.1395
6	16.904	18.121	19.558	21.466	23.813	26.444	29.723	-1.1048	0.1454
6.5	17.748	19.055	20.611	22.699	25.304	28.274	32.058	-1.1562	0.1513
7	18.607	20.012	21.696	23.976	26.856	30.194	34.531	-1.1902	0.1573
7.5	19.495	21.005	22.826	25.312	28.486	32.213	37.144	-1.2073	0.1632
8	20.426	22.051	24.021	26.726	30.211	34.349	39.903	-1.2091	0.1689
8.5	21.411	23.162	25.292	28.233	32.047	36.615	42.811	-1.1970	0.1743
9	22.459	24.348	26.653	29.847	34.010	39.023	45.871	-1.1729	0.1795
9.5	23.575	25.615	28.109	31.575	36.105	41.577	49.077	-1.1392	0.1843
10	24.763	26.967	29.666	33.422	38.336	44.278	52.422	-1.0983	0.1888
10.5	26.026	28.408	31.327	35.390	40.705	47.123	55.894	-1.0520	0.1929
11	27.372	29.946	33.099	37.486	43.214	50.108	59.477	-1.0018	0.1965
11.5	28.821	31.599	35.000	39.724	45.872	53.234	63.157	-0.9493	0.1994
12	30.398	33.392	37.052	42.121	48.689	56.500	66.928	-0.8973	0.2015
12.5	32.136	35.352	39.275	44.690	51.669	59.907	70.790	-0.8504	0.2024
13	34.053	37.490	41.672	47.423	54.793	63.427	74.717	-0.8131	0.2021
13.5	36.145	39.793	44.218	50.279	58.005	66.995	78.647	-0.7887	0.2004
14	38.380	42.216	46.857	53.192	61.228	70.524	82.487	-0.7791	0.1976
14.5	40.696	44.691	49.512	56.070	64.359	73.903	86.122	-0.7846	0.1937
15	43.021	47.141	52.101	58.831	67.311	77.043	89.461	-0.8033	0.1892
15.5	45.276	49.487	54.548	61.399	70.010	79.873	92.430	-0.8322	0.1843
16	47.388	51.662	56.788	63.716	72.407	82.346	94.986	-0.8669	0.1795
16.5	49.294	53.607	58.772	65.741	74.470	84.440	97.110	-0.9029	0.1748
17	50.955	55.292	60.478	67.466	76.207	86.180	98.845	-0.9371	0.1707
17.5	52.351	56.704	61.903	68.901	77.644	87.610	100.261	-0.9668	0.1673
18	53.483	57.850	63.062	70.071	78.822	88.792	101.443	-0.9908	0.1647
18.5	54.365	58.750	63.981	71.013	79.790	89.788	102.477	-1.0085	0.1630
19	55.031	59.439	64.698	71.767	80.593	90.653	103.431	-1.0214	0.1622
19.5	55.529	59.965	65.260	72.382	81.282	91.438	104.359	-1.0309	0.1621
20	55.920	60.388	65.725	72.911	81.904	92.185	105.297	-1.0391	0.1624

*50th percentile = model parameter M

Table S7: Smoothed **weight (kg) for height (cm)** percentiles and model parameters for **girls.**

	Percentiles							Model parameters	
**height**	**3**	**10**	**25**	**50***	**75**	**90**	**97**	**L**	**S**
50	2.876	3.054	3.240	3.456	3.679	3.888	4.100	0.3954	0.0941
55	3.757	3.978	4.212	4.485	4.772	5.043	5.322	0.1959	0.0926
60	4.823	5.090	5.376	5.712	6.069	6.410	6.765	0.0008	0.0900
65	5.954	6.265	6.599	6.995	7.420	7.828	8.258	-0.1859	0.0869
70	7.034	7.382	7.760	8.210	8.697	9.170	9.671	-0.3628	0.0845
75	8.034	8.417	8.834	9.335	9.882	10.417	10.990	-0.5330	0.0831
80	8.998	9.416	9.873	10.427	11.037	11.639	12.290	-0.6999	0.0825
85	9.996	10.450	10.951	11.563	12.241	12.919	13.659	-0.8645	0.0825
90	11.049	11.543	12.091	12.765	13.520	14.281	15.123	-1.0243	0.0827
95	12.148	12.685	13.284	14.026	14.865	15.720	16.676	-1.1735	0.0833
100	13.275	13.861	14.519	15.341	16.278	17.244	18.337	-1.3047	0.0846
105	14.459	15.108	15.843	16.769	17.837	18.952	20.234	-1.4167	0.0877
110	15.733	16.468	17.308	18.378	19.631	20.962	22.522	-1.5106	0.0931
115	17.113	17.960	18.937	20.201	21.709	23.346	25.317	-1.5861	0.1009
120	18.599	19.580	20.726	22.232	24.064	26.101	28.627	-1.6329	0.1101
125	20.196	21.332	22.672	24.456	26.666	29.176	32.378	-1.6415	0.1196
130	21.945	23.253	24.808	26.897	29.517	32.540	36.470	-1.6078	0.1280
135	23.956	25.465	27.266	29.698	32.769	36.339	41.025	-1.5378	0.1354
140	26.260	28.011	30.106	32.944	36.539	40.732	46.250	-1.4429	0.1426
145	28.837	30.880	33.331	36.661	40.891	45.835	52.350	-1.3411	0.1506
150	31.930	34.305	37.161	41.048	45.996	51.788	59.427	-1.2625	0.1572
155	35.696	38.397	41.646	46.068	51.693	58.271	66.929	-1.2284	0.1593
160	39.964	42.922	46.472	51.289	57.392	64.495	73.790	-1.2441	0.1556
165	44.089	47.228	50.984	56.065	62.481	69.920	79.617	-1.2949	0.1499
170	47.730	51.003	54.917	60.207	66.884	74.629	84.739	-1.3634	0.1453
175	51.037	54.444	58.520	64.039	71.026	79.168	89.871	-1.4365	0.1427
180	54.375	57.923	62.178	67.954	75.300	83.918	95.360	-1.5125	0.1410

*50th percentile = model parameter M

Table S8: Smoothed **weight (kg) for height (cm)** percentiles and model parameters for **boys.**

	Percentiles							Model parameters	
**height**	**3**	**10**	**25**	**50***	**75**	**90**	**97**	**L**	**S**
50	2.876	3.049	3.233	3.445	3.666	3.873	4.086	0.3207	0.0933
55	3.759	3.987	4.227	4.507	4.799	5.073	5.355	0.2847	0.0940
60	4.828	5.115	5.418	5.772	6.142	6.491	6.850	0.2372	0.0930
65	5.968	6.309	6.670	7.091	7.534	7.952	8.384	0.1699	0.0903
70	7.088	7.473	7.882	8.362	8.868	9.348	9.845	0.0750	0.0874
75	8.145	8.567	9.019	9.550	10.114	10.652	11.212	-0.0475	0.0850
80	9.164	9.623	10.117	10.701	11.325	11.925	12.554	-0.1903	0.0837
85	10.178	10.675	11.213	11.854	12.546	13.215	13.924	-0.3478	0.0832

height	Percentiles							Model parameters	
	3	10	25	50*	75	90	97	L	S
90	11.213	11.750	12.334	13.037	13.802	14.551	15.352	-0.5192	0.0833
95	12.276	12.851	13.483	14.248	15.092	15.927	16.832	-0.7107	0.0836
100	13.413	14.026	14.706	15.539	16.468	17.401	18.427	-0.9273	0.0838
105	14.676	15.335	16.072	16.986	18.021	19.078	20.262	-1.1667	0.0847
110	16.044	16.767	17.584	18.613	19.800	21.039	22.463	-1.4082	0.0878
115	17.475	18.285	19.213	20.405	21.813	23.324	25.118	-1.6277	0.0937
120	18.943	19.861	20.930	22.330	24.029	25.912	28.243	-1.8025	0.1018
125	20.526	21.568	22.799	24.439	26.479	28.813	31.826	-1.9183	0.1102
130	22.343	23.523	24.930	26.830	29.236	32.057	35.818	-1.9691	0.1172
135	24.466	25.810	27.421	29.615	32.425	35.768	40.320	-1.9590	0.1231
140	26.830	28.374	30.233	32.779	36.065	40.013	45.458	-1.8979	0.1296
145	29.293	31.086	33.254	36.240	40.123	44.827	51.387	-1.7996	0.1379
150	31.893	33.987	36.532	40.054	44.665	50.293	58.211	-1.6806	0.1476
155	34.829	37.273	40.253	44.392	49.829	56.485	65.867	-1.5572	0.1568
160	38.432	41.261	44.709	49.491	55.754	63.378	74.012	-1.4438	0.1623
165	42.773	45.987	49.888	55.268	62.245	70.621	82.061	-1.3513	0.1628
170	47.604	51.164	55.459	61.330	68.846	77.714	89.533	-1.2869	0.1593
175	52.265	56.114	60.731	66.994	74.927	84.160	96.241	-1.2527	0.1548
180	56.307	60.395	65.286	71.893	80.221	89.848	102.339	-1.2463	0.1519
185	59.627	63.935	69.089	76.058	84.850	95.028	108.260	-1.2616	0.1515
190	62.498	67.021	72.449	79.815	89.157	100.048	114.342	-1.2909	0.1529
195	65.195	69.940	75.656	83.456	93.425	105.167	120.795	-1.3275	0.1554
200	67.878	72.852	78.869	87.130	97.782	110.479	127.656	-1.3665	0.1582

*50th percentile = model parameter M

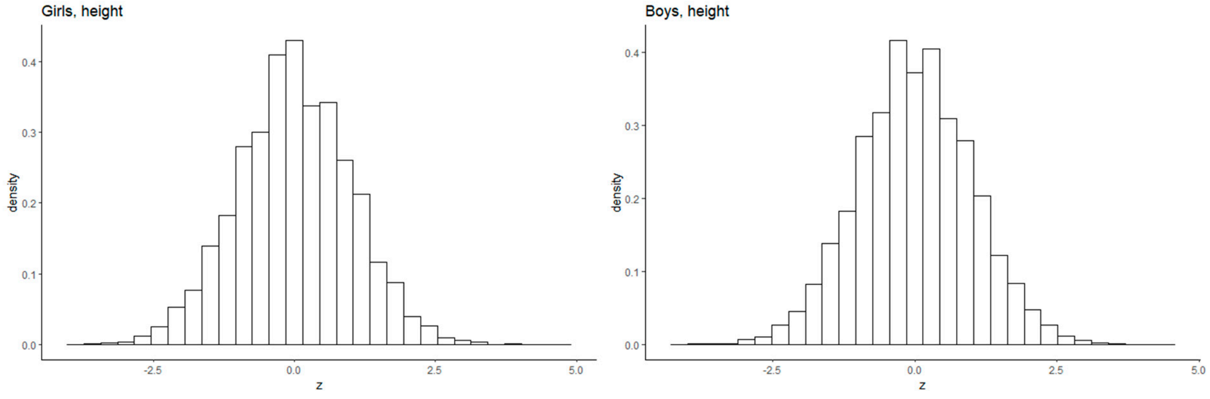

Figure S1: Density histograms of height data.

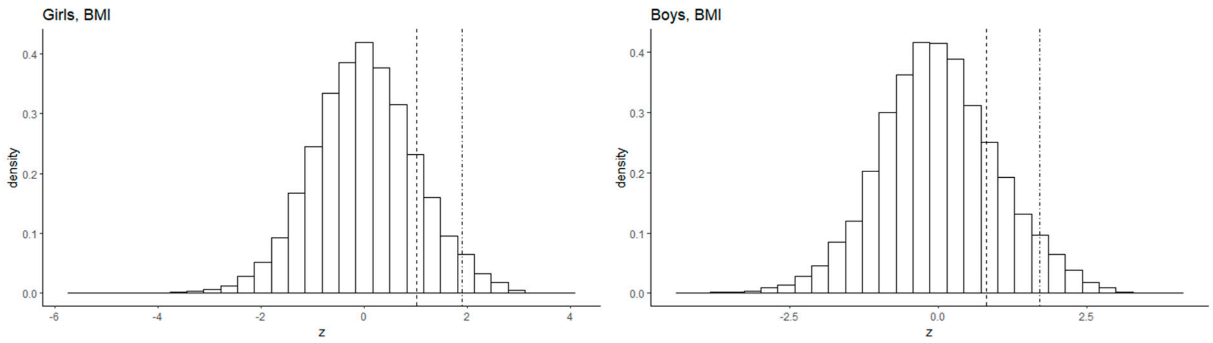

Figure S2: Density histograms of BMI data. Dashed line: calculated cut-off for overweight, dot-dashed line: calculated cut-off for obesity (see section 3.3).

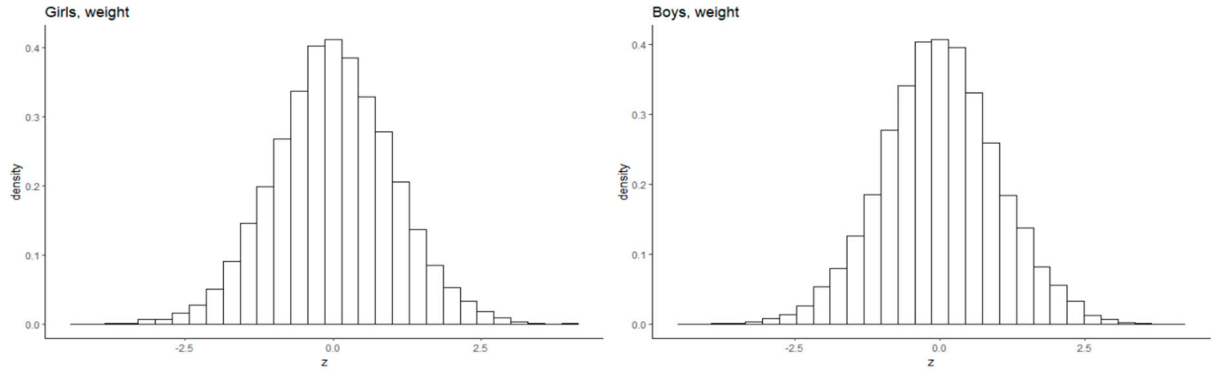

Figure S3: Density histograms of weight data.
