# Supplementary material for: Updated Swiss Growth References 2025: No Height Differences, but BMI Variations Associated with Migration
Source: J Clin Med. 2025 Aug 21;14(16):5912. doi: 10.3390/jcm14165912 (PMC12387566; doi:10.3390/jcm14165912)
Supplement: Supplementary file 1 [file jcm-14-05912-s001.zip › jcm-3796415 Supplemental material to Manuscript Cohort 2019+2025 Supp. Text S1-S4. (Edited).pdf]

Supplemental Material, Text S1–S4

Supplementary Text S1

Participating Pediatric Practices—German-speaking central region (Data Collection 2019)
We thank the following pediatricians and practices for their participation in the 2019 data collection:
Dr. K. Altmann (Rüti), Dr. D. Baiao Picciati (Küssnacht), Dr. I. Baruffol (Thalwil), Dr. C. Baumgartner (Gossau), Dr. M. Belvedere (Zürich), Dr. Y. Bestmann (Wetzikon), Dr. A. Bewer (Thalwil), Dr. M. Bischofsberger (Zumikon), Dr. S. Braunschweig (Jona), Dr. J. Cahlic (Affoltern am Albis), Dr. C. Clerc (Egg), Dr. S. Crameri (Binningen), Dr. J. Crone (Pfannenstiel), Dr. P. Cronin (Wil), Dr. F. D'Abbraccio (Zürich), Dr. S. Dübendorfer (Tägerwilen), Dr. C. T. Eberhardt (Wallisellen), Dr. M. Fust Aguilera (Wil), Dr. T. Gallmann (Zürich), Dr. M. Girsberger (Frauenfeld), Dr. V. Griebel (Rapperswil-Jona), Dr. L. Hochstrasser (Zollikon), Dr. A. Hugi Maier (Frauenfeld), Dr. K. Imahorn (Wil SG), Dr. B. Jäppinen (Wil SG), Dr. P. Kapassakis (Thalwil), Dr. R. Kehrt (Meilen), Dr. S. Köppelmann (Thalwil), Dr. M. Landolt, Binningen; Dr. A. Locher (Stäfa), Dr. M. Malosti (Jona), Dr. T. Marti (Näfels), Dr. J. Meyer Menzi (Zürich), Dr. C. Moran (Jona), Dr. T. Moser (Bülach), Dr. T. Murer (Lachen), Dr. J. Niederer-Pelzer (Dielsdorf), Dr. P. Orban (Dübendorf), Dr. C. Peters (Frauenfeld), Dr. C. Pingoud (Wallisellen), Dr. A. Rostetter (Schwamendingen), Dr. S. Rupp (Einsiedeln), Dr. S. Schadde (Wetzikon), Dr. M. Schenker (Wallisellen), P. Schibler (Stäfa), A. Schneider (Zürich), S. Schönbeck (Zürich), R. Schöpke (Frauenfeld), Dr. R. Schramedei (Laachen), Dr. H. Schütze (Zürich), Dr. T. Schwank (Thalwil), Dr. C. Solèr-Bischof (Wil SG), Dr. S. Strunz (Zollikon), Dr. H. Ubieto (Gossau SG), Dr. A. Vogt (Rüti), Dr. S. Wegner (Egg b. Zürich), Dr. C. Wilhelm (Thalwil), Dr. O. Zerwetz (Egg), Dr. S. Züllig Naef (Zürich).

Supplementary Text S2

Participating Educational and Public Health Institutions (Data Collection 2019)
We also thank the following institutions for their cooperation:
Allgemeine Berufsschule Zürich, Baugewerbliche Berufsschule Zürich, Berufsbildungsschule Winterthur, Berufsschule Aarau, Berufsschule für Detailhandel Zürich, Berufsschule für Mode und Gestaltung, Gymnasium Hohe Promenade, Gymnasium Unterstrass, Kantonsschule Freudenberg, Kantonsschule Oerlikon Nord, Kantonsschule Stadelhofen, Primarschule Fluntern, Primarschule Hasenbühl, Primarschule Richterswil, Primarschule Steiacher Brüttisellen, Realgymnasium Rämibühl, Schule Herzogenmühle, Schule Oescher Zollikon, Schulhaus Rüterwis Zollikerberg, Schule Triemli, Schule Zurlinden, Schulthess Klinik, Strickhof, Villa Kunterbunt Letzigraben, Zentrum für Ausbildung im Gesundheitswesen, Schulgesundheitsdienste der Stadt Zürich, Eidgenössisches Departement für Verteidigung, Bevölkerungsschutz und Sport (A. Stab, Sanität), Swiss National Cohort, Federal Statistical Office.

Supplementary Text S3

Participating Pediatric Practices—French-speaking western region (Romandie)
We thank the following pediatricians and group practices and clinics across French-speaking Switzerland for their participation in the extended data collection:
Cabinet de groupe Dr. Raymond Bertrand, Dr. Christina Exhenry (Genève); Cabinet de groupe Dr. Marie-Noelle Biesel-Desthieux, Dr. Sylvie Brouze Guilbert (Chêne-Bourg); Cabinet de groupe Dr. Anais Mialon, Dr. Reinhardt Owlya (Lausanne); Cabinet de pédiatrie Clément, Dr. Chiara Giracasa, Dr. Yvonne Bérard (Ecublens); Cabinet de pédiatrie du Faubourg, Jaquet-Pilloud, Dr. Timothy Hirter; Dr. Camille Lorenzana, Dr. Dario Rodriguez (Neuchâtel); Cabinet de pédiatrie La Planta, Dr. Selina Pinösch, Dr. Jean-Yves Corajod (Cologny); Cabinet Dr. Catherine Bourban (Marin); Cabinet médical La Tour, Dr. Valérie Dénervaud, Dr. Ines Goglund (La Tour-de-Trême); Cabinet pédiatrique Dr. Elisabeth Lopes (Estavayer-le-Lac); Centre Médical Charmilles, Dr. Toulou Abah (Genève); Centre médical de la Chapelle, Dr. Tido von Schoen-Angerer, Dr. Biro Levescot (Onex); Centre pédiatrique Dr. Terry Mizrahi, Dr. Jeff Huser, Dr. Vreni Boss (Cheseaux-sur-Lausanne); Dr. Anne Tornay (Martigny); Dr. Avdilova Ibraimi (Cointrin); Dr.

Daniel Halpérin, Dr. Shahar Picard-Trabelsi (Genève); Dr. Françoise Riand (Martigny); Dr. Marie Claire Gaillard (Martigny); Dr. Thomas Gehrke (Hôpital de Martigny); Dr. Jean-Michel Poncet (Monthey); Dr. Lucia Romeo (Verbier); Dr. Revaz-Kuchler (Nyon); Dr.Ignacio Rimbau (Martigny); Dr. Anne Romanowicz (Meyrin); Dr. Anne Ruffieux-Jordan (Plan-les-Ouates); Dr. Simon Fluri (Visp); Dr. Vincent Genoud (Genève); Dr. Véronique Isabelle Widmeier, Dr. Marie-José Matthey-de-Perrot (Faubourg du Lac); Groupe Médical des Avanchets, Dr. Muriel Piller Claramunt (Les Avanchets); Groupe Médical du Petit-Saconnex SA, Dr. Philippe Desmangles-Simonet (Genève); La Toula Pédiatrie, Dr. Stéphanie Gachet, Dr. Isabelle Osinga, Dr. Diana Liard (Bulle); Le Cabinet (Cossonay-Ville); PediaBulle, Isea Pena, De Lucia, Bernal, Dr Roger Sanmiquel Safont. (Bulle); Dr. Laure Ziegler, FMH Pädiatrie, Genève; Praxis Kinderwelt Dr. med Julia Ambühl, Dr. med. Peter Luggen (Brig-Glis); Dr. Caroline Hefti-Rütsche, Yverdon-les-Bains.

We sincerely thank Dr. med. Josef Laimbacher for his commitment to the national reference curve project and his key role in initiating and coordinating the extended data collection in 2025.

Supplementary Text S4

Participating Pediatric Practices—Italian-speaking southern region (Ticino)

We thank the following pediatricians and practices in the Italian-speaking region for their contributions to the extended data collection:

Dr. Enrico Capuano, Dr. Cristina Massai (Mendrisiotto); Dr. Claudio Roduit (Pregassona); Dr. Lorenzo Bianchetti (Locarno); Dr. Luca Pissoglio (Locarno); Dr. Nicola Fratini (Giubiasco);
Dr. Dina Tognini, Dr. Chiara Roncoroni (Massagno); Dr. Alexandra Sozzo (Bellinzona);
Dr. Daniela Pedrazzini (Paradiso); Dr.ssa Giovanna Ferrazzini (Mendrisio);
Dr. Patrizia Tessiatore (Lamone).
Special thanks go to Prof. Dr. Gian Paolo Ramelli for his support in organizing data collection in the Ticino region.

Institutional Support

We gratefully acknowledge the organizational assistance provided by Claudia Baeriswyl and Mélissande Imseng from Pädiatrie Schweiz during the extended data collection in 2025.
